# Synergistic therapeutic impact of dichloroacetate nanoparticles and doxorubicin in modulating pyruvate dehydrogenase kinase in breast carcinoma model

**DOI:** 10.1038/s41598-025-34562-7

**Published:** 2026-01-23

**Authors:** Maha M. Salem, Amira T. Khattab, Doha M. Beltagy, Mai M. El-Keiy

**Affiliations:** 1 Biochemistry Division, Chemistry Department, Faculty of Science, Tanta University, Tanta, 31257 Egypt; 2 Biochemistry Department, Faculty of Science, Damanhour University, Damnhour, 22514 Egypt

**Keywords:** Dichloroacetate nanoparticles, Pyruvate dehydrogenase kinase, Breast cancer, Mitochondrial membrane, Cancer metabolism, Cancer, Drug discovery, Oncology

## Abstract

Breast cancer is treated with chemotherapies causing severe organ side effects. Increased pyruvate dehydrogenase kinase (PDK) enzymes in cancer cell metabolism lead to tumor resistance. This study examined dichloroacetate nanoparticles (DCA-PNPs)/doxorubicin (Dox) impact on PDK enzymes in Ehrlich ascites carcinoma (EAC) cells. DCA-PNPs were synthesized using poly D, L-lactic-co-glycolide, polyvinyl alcohol, and characterized by encapsulation efficiency, drug-loading capacity, spectroscopy, and microscopy. Molecular docking and ADMET analysis were conducted. Seventy female CD1 mice were divided into 10 groups (n = 7). (Gp1) was normal negative control. Gp2 to Gp4 received DCA (50 mg/kg), DCA-PNPs (50 mg/kg), and Dox (0.2 mg/kg), intraperitoneal (i.p.) injection. Gp5 to Gp10 were inoculated with EAC cells 0.5 × 10^6^ /mouse. GP5 were untreated group, served as a positive control (EAC-untreated mice or EAC-bearing mice group). GP6 to GP10 were treated with Dox, DCA, DCA-PNPs, Dox/DCA, and Dox/DCA-PNPs (i.p.). On day 14, tumor profile, molecular analysis, and hepatorenal alterations were assessed. Results showed DCA-PNPs size was 22.5 ± 1.72 nm, and (ζ) was − 9.5 mV. Dox exhibited strongest binding affinity across PDKs (− 7.7 to − 8.3 kcal/mol), while DCA showed modest affinities (− 3.7 to − 4.0 kcal/mol). Dox/DCA-PNPs treatment decreased tumor profile and hepatorenal alterations with PDKs gene suppression, increased cancer cell apoptosis and cycle arrest at G0/G1 phase (76.1 and 64.8%). Dox/DCA-PNPs demonstrated anti-tumor activity via inhibiting PDK enzyme.

**Supplementary Information:**

The online version contains supplementary material available at 10.1038/s41598-025-34562-7.

## Introduction

Globally, among different cancer types, breast cancer (BC) poses a significant global health threat with increases in mortality rate^1^. Particularly in Egypt, BC showed an increase in incidence and mortality rates^2^. During the cancer metabolic pathway, the Warburg effect increased glycolysis, decreased oxidative phosphorylation, and promoted cancer cell progression^3^. Pyruvate dehydrogenase kinase (PDK) control cellular metabolism by inactivating pyruvate dehydrogenase complex (PDC)^4^. The PDK family comprises four distinct isoforms (PDK1, 2, 3 and 4), which are differentially regulated in response to various physiological and pathological conditions, including cancer^5^. PDK enzymes upregulated the Warburg effect in cancer cells metabolic pathway^6^. Furthermore, PDK enzymes assists cancer cells to maintain redox balance and resist oxidative stress^7^. Multiple factors affect PDKs mechanism, such as heightened glycolysis, lactate synthesis, and glutaminolysis, alongside diminished mitochondrial oxidative phosphorylation, pyruvate oxidation, and glucose oxidation^8^. The signalling of hypoxia-inducible factor 1α (HIF-1α) enhances PDK enzyme activity^9^.

The Ehrlich ascites carcinoma (EAC) model is employed to investigate breast cancer biology and assess prospective therapeutics in female mice^10^. The EAC model, derived from a mammary adenocarcinoma, serves as a transplantable tumour model for breast cancer in vivo. It shares pathological features with human breast cancer, including rapid proliferation, high mitotic activity, metabolic reprogramming, and chemotherapeutic responsiveness. Its rapid growth and reproducibility make EAC suitable for evaluating anticancer therapeutics^10^. Prior research employed the EAC model to assess the effectiveness of various nanoparticle (NP) treatments^11–14^. Doxorubicin (Dox) is an FDA-sanctioned chemotherapeutic agent. It utilised in the treatment of solid tumors, such as ovarian, breast, and gastrointestinal malignancies^15^. This anti-cancer medication has multiple adverse effects, including allergic responses, cardiac impairment, alopecia, bone marrow suppression, emesis, and bladder irritation^16^. Various tactics have been employed to improve the efficacy and safety profile of DOX, including liposomal encapsulation, nanoparticle delivery systems, combination therapy, cardioprotective drugs, and metabolic regulators^17^. Dichloroacetate (DCA) is recognised as a metabolic regulator^18^. It inhibits PDK, facilitating a transition from glycolysis to oxidative phosphorylation in neoplastic cells^19^. DCA inhibits PDKs activity by binding to its N-terminal regulatory domain, cause conformational switch that inactivates PDKs, resulting in the dephosphorylation and activation of the PDC, promoting pyruvate to oxidize decarboxylation to acetyl-CoA and enhancing mitochondrial glucose oxidation^20^.

Nanoparticles (NPs) can be produced using many ways, including the emulsion/evaporation method^21^. Treatment with NPs improved drug efficacy and reduced damage to healthy tissues^22^. This system enhance drug solubility, targeted delivery, controlled release mechanisms, improving bioavailability and minimizing side effects^23^.

Recent studies have highlighted the importance of combining chemotherapeutic agents with naturally occurring flavonoids to enhance anticancer efficacy and overcome drug resistance. Baicalein-based nano-delivery systems, in particular, have demonstrated improved stability, cellular uptake, and synergistic therapeutic activity in breast cancer models. For example, a ratiometric codelivery nano emulsion incorporating paclitaxel and baicalein showed significant enhancement in tumor inhibition and apoptotic signaling pathways in breast carcinoma models. This supports the rationale for developing dual-drug nanoformulations to potentiate anticancer responses^24^.

Although numerous studies have explored either DCA or Dox delivered via nanoparticles, most of these focused primarily on improving pharmacokinetics, delivery efficiency, or toxicity profiles. Few have addressed the metabolic reprogramming aspect of cancer therapy, particularly the direct inhibition of PDK enzymes, which are central to cancer metabolic adaptation. Furthermore, to the best of our knowledge, no previous study has comprehensively investigated the combined use of DCA-loaded nanoparticles and Dox to achieve synergistic anticancer effects through simultaneous metabolic and chemotherapeutic targeting in the EAC model. Therefore, this study was designed to fill this critical gap. We first evaluated the molecular docking interactions between DCA and various PDK isoforms to assess their binding affinity and inhibitory potential. Then examined the synergistic impact of a combined treatment using DCA-PNPs and Dox on the suppression of PDK enzymes and metabolic reprogramming in EAC-bearing mice. This integrated approach aims to enhance therapeutic efficacy while potentially reducing systemic toxicity, offering a new strategy for combines metabolic modulation with conventional chemotherapy for breast cancer treatment.

## Results

### Nanoparticles characterizations

#### Encapsulation efficacy %, and drug loading capacity % of DCA-PNPs

**Fig. 1:**
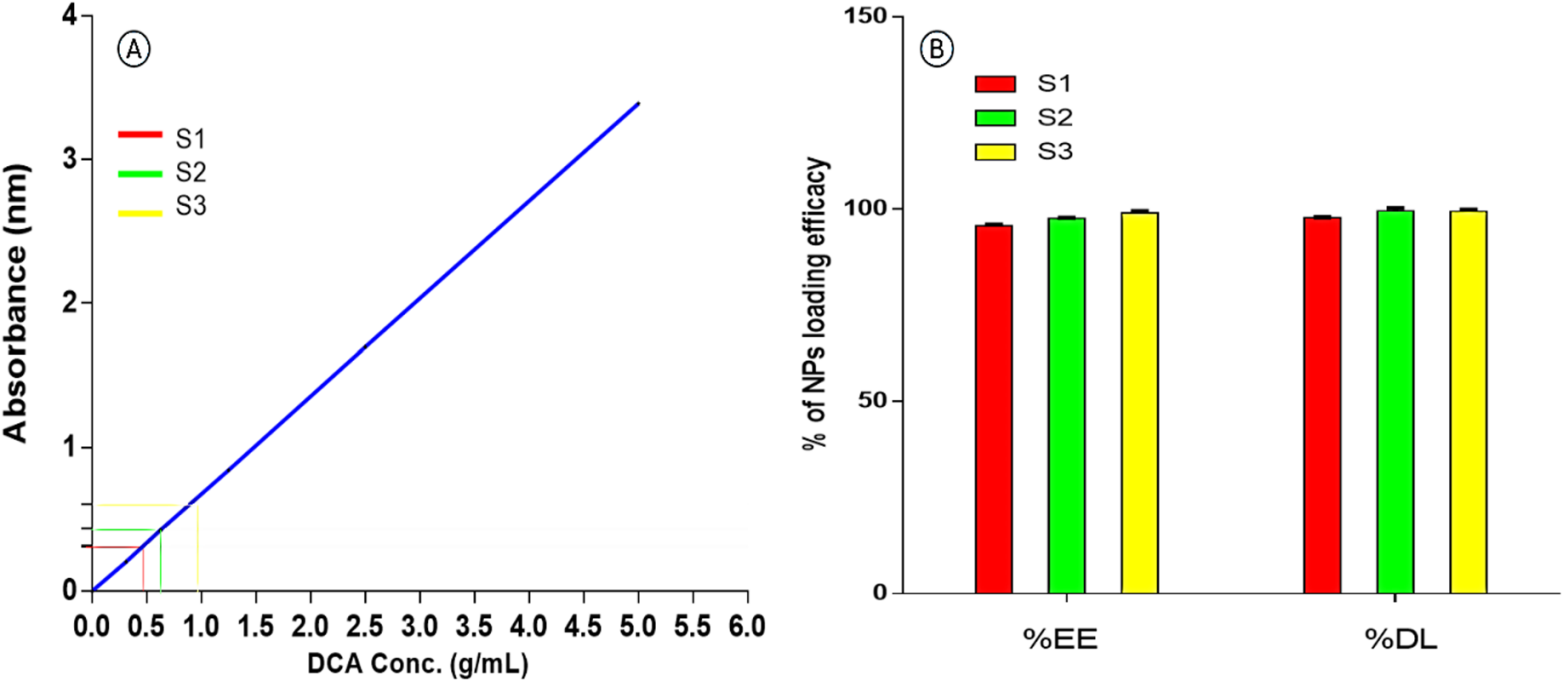
(**A**) DCA-PNPs linear calibration curve, where the linear equation was (y = 0.67896x − 0.00357) with a correlation coefficient of (R^2^ = 0.9), indicating excellent linearity. (**B**) the %EE and %DL of DCA-PNPs for three samples (S1, S2, and S3).

The encapsulation efficacy %EE , and Drug loading %DL capacity were determined based on the calibration curve of DCA-PNPs with 3 samples (S1, S2, and S3) with concentration of (0.45, 0.625, and 0.885 gm/mL), respectively as shown in (Fig. 1A), the %EE was 97.43 ± 0.95% and %DL was 98.9 ± 0.55% (Fig. 1B).

#### Transmission and scanning electron microscopy

**Fig. 2:**
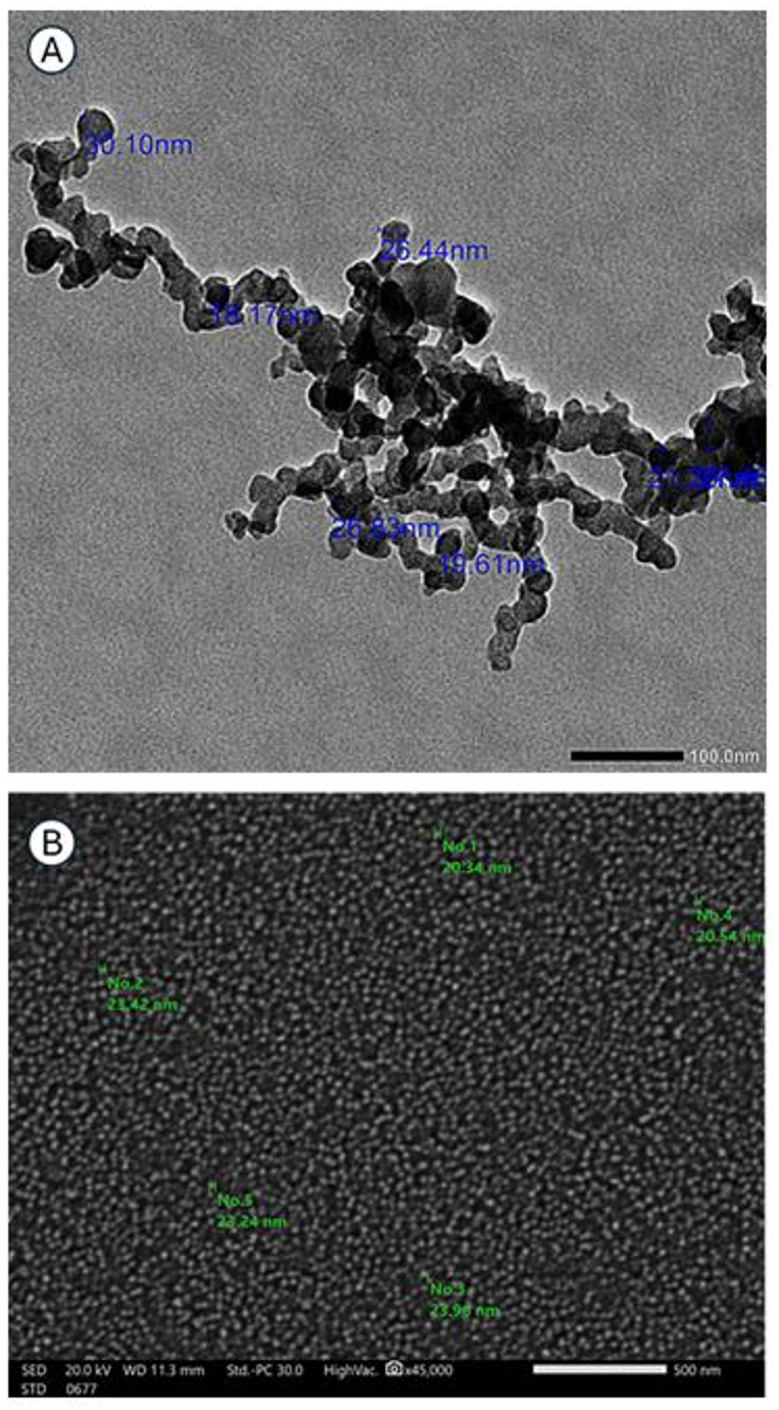
(**A**) SEM micrograph depicting the surface characteristics of DCA-PNPs, (**B**) TEM micrograph showing DCA-PNPs size.

SEM revealed that DCA-PNPs exhibited a round shape with a mean size of 22.5 ± 1.72 nm and distributed in a single layer. Although some clustering and irregular configurations were observed, such aggregation is frequently reported during SEM imaging of polymeric nanoparticles and is often attributed to residual stabilizers such as polyvinyl alcohol (PVA), which can increase solution viscosity (Fig. 2A). TEM verified that DCA-PNPs exhibited a more defined structural arrangement, appearing as spheres with smooth surfaces and diameters ranging from 15 to 30 nm, although slight image clarity may be related to sample thickness or electron beam scattering, where electrons may interact more with the sample, leading to both inelastic scattering and a reduction in the number of transmitted electrons (Fig. 2B).

#### Ultraviolet and Fourier transform infrared spectral analyses

**Fig. 3:**
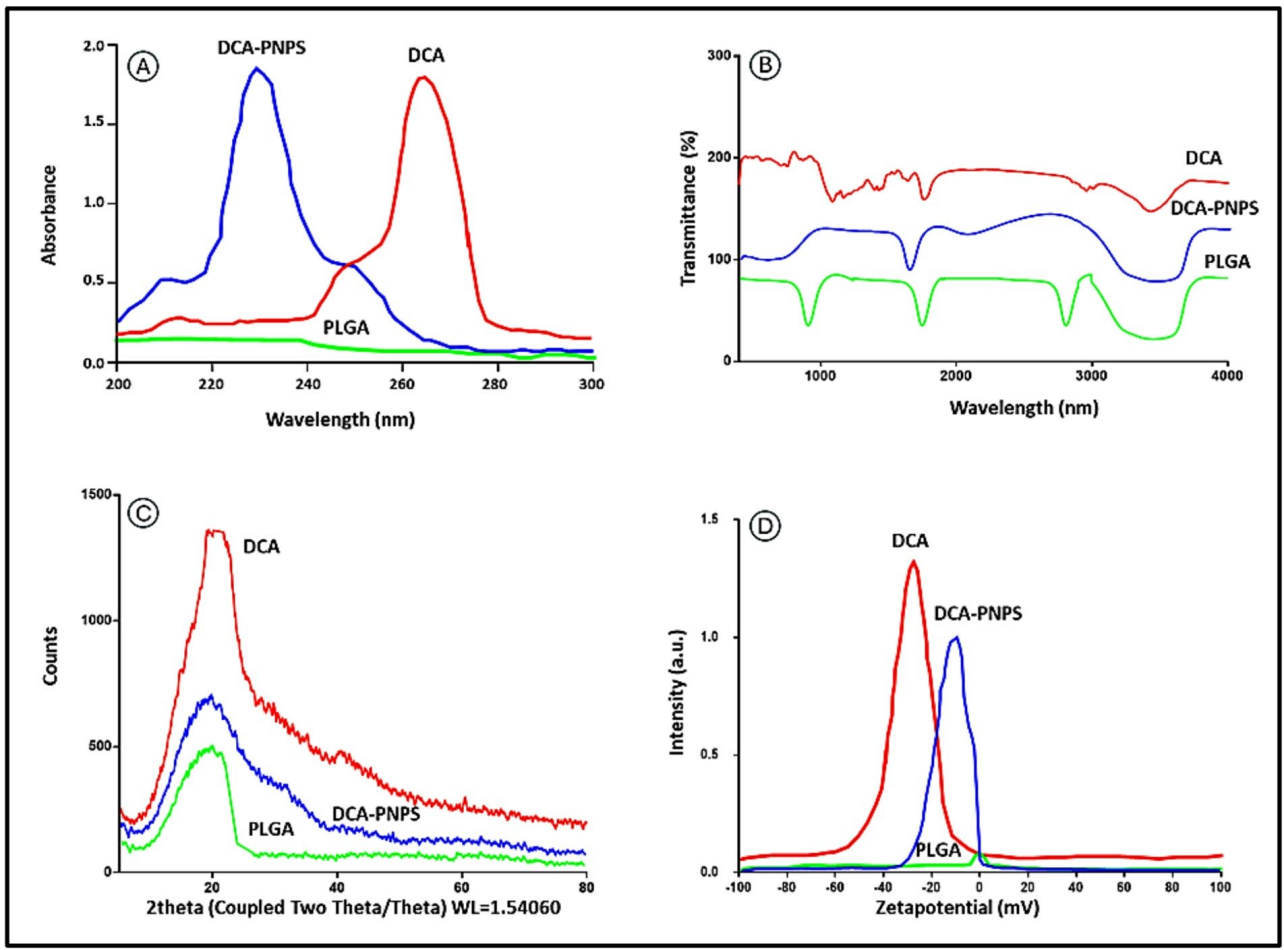
DCA, DCA-PNPs, and PLGA [(**A**) UV spectrum; (**B**) FTIR spectrums; (**C**) XRD patterns; (**D**) zeta-potential].

The results obtained from UV spectrum of DCA and DCA-PNPs exhibited a distinctive strong absorption peak at (265 nm due to C–Cl/C = O bonds**,** and 230 nm as a shifted peak)**,** respectively. In contrast PLGA showed a broad weak peak at 214 nm) as it usually transparent at this region (Fig. 3A).

FTIR diffractometer analyzed the interaction between PLGA and DCA during encapsulation by identifying their functional groups. That DCA showed bands at 891 cm^−1^ donating C–Cl stretching, 1193 cm^−1^ indicated C–H bond, 1727 cm^−1^ for C = O group, and 2791 cm^−1^ indicated C–H vibration. Furthermore, PLGA showed (498, 1167, and 2952) cm^-1^ bands donating for C–C, C–O, and C–H stretching, respectively. The peaks at (1641, and 1757) cm^−1^ were determined C = O group within the compound. While DCA-PNPs showed bands at 415 and 593 cm^−1^, denoting C–C as well as C–O expanding vibrations. The peak at 1683 cm^−1^ indicated the presence of the C = O group, whilst the band at 3438 cm^−1^ was associated with the stretching vibration of the OH group (Fig. 3B).

#### X-ray diffraction patterns

DCA exhibited distinctive and sharp peak in XRD pattern at (2θ = 21.23°) indicated its nature crystalline, while DCA-PNPs showed broad and low intense peak at (19.032°) indicated its encapsulation. In contrast PLGA revealed a broad halo amorphous peak at (19.545°) indicated its crystalline. All peaks at 100% intensity, attributed to DCA/PLGA interactions (Fig. 3C).

#### Zeta-potential

The potential of DCA, DCA-PNPs, and PLGA with (1%) concentrations were measured, and the anionic characteristics of these had a negative (−ve) zeta potential represented as (zero, −9.5 mV, −30 mV), (Fig. 3D).

### Molecular docking

**Fig. 4:**
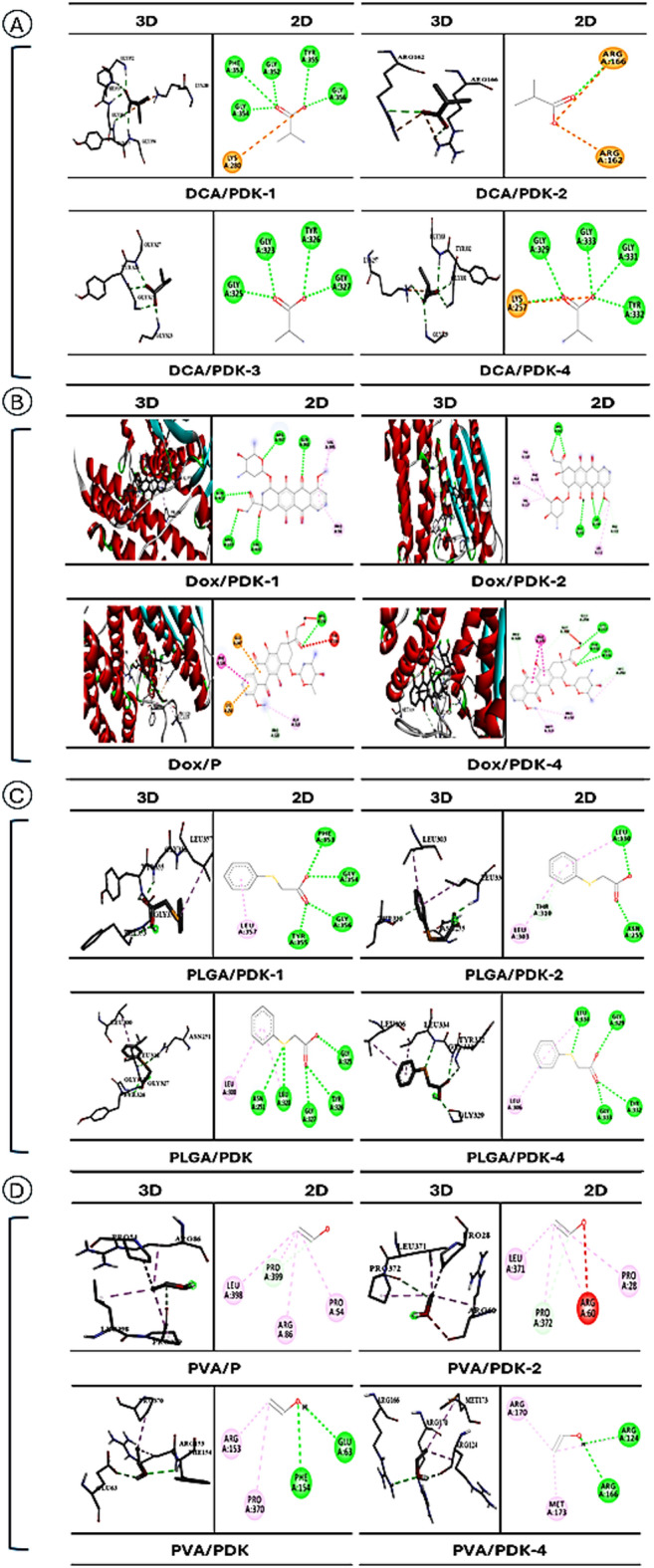
3D and 2D structures of PDKs enzyme-ligand complex interaction at catalytic regions with (**A**) DCA, (**B**) Dox, (**C**) PLGA, and (**D**) PVA.

The docking data and ADMET analysis indicated the binding interactions between four PDKs and the studied ligands (DCA, Dox, PLGA, and PVA). Where docking showed that Dox consistently showed the strongest binding affinity across all PDKs, with scores ranging from − 7.7 to − 8.3 kcal/mol. Additionally, DCA showed modest binding affinities (− 3.7 to − 4.0 kcal/mol) across all PDKs. the polymeric compounds PLGA and PVA showed intermediate and weak binding affinities respectively, with PLGA around − 5.4 kcal/mol and PVA around − 2.8 kcal/mol. Collectively, all 4 ligands notably had excellent interaction with PDK1 and PDK4. Furthermore, DCA forming stable 5 H-B with key residues consisting of [GLY352, GLY354, GLY356, PHE353, and TYR355]. As well Dox formed 5 conventional hydrogen bonds (H-B) with key residues including [ARG183, GLN368, ARG397, VAL400, and ASN402]. While PLGA and PVA formed 3 and 1 H-B at their active sites respectively, [PHE353, GLY354, GLY356, and TYR355 for PLGA] and [PRO399 for PVA], as shown in (Table 1, and Fig. 4).

**Table 1:** The calculated molecular docking scores (kcal/mol).

Receptors	PDK1-2Q8H				PDK2-4MPN				PDK3-1Y8O				PDK4-7EBB			
Ligands	DCA	Dox	PLGA	PVA	DCA	Dox	PLGA	PVA	DCA	Dox	PLGA	PVA	DCA	Dox	PLGA	PVA
DS	− 4	− 8.3	− 5.5	− 2.9	− 3.9	− 7.9	− 5.3	− 2.7	− 3.8	− 7.7	− 5.3	− 2.6	− 3.7	− 8	− 5.4	− 2.8

### ADMET analysis

**Fig. 5:**
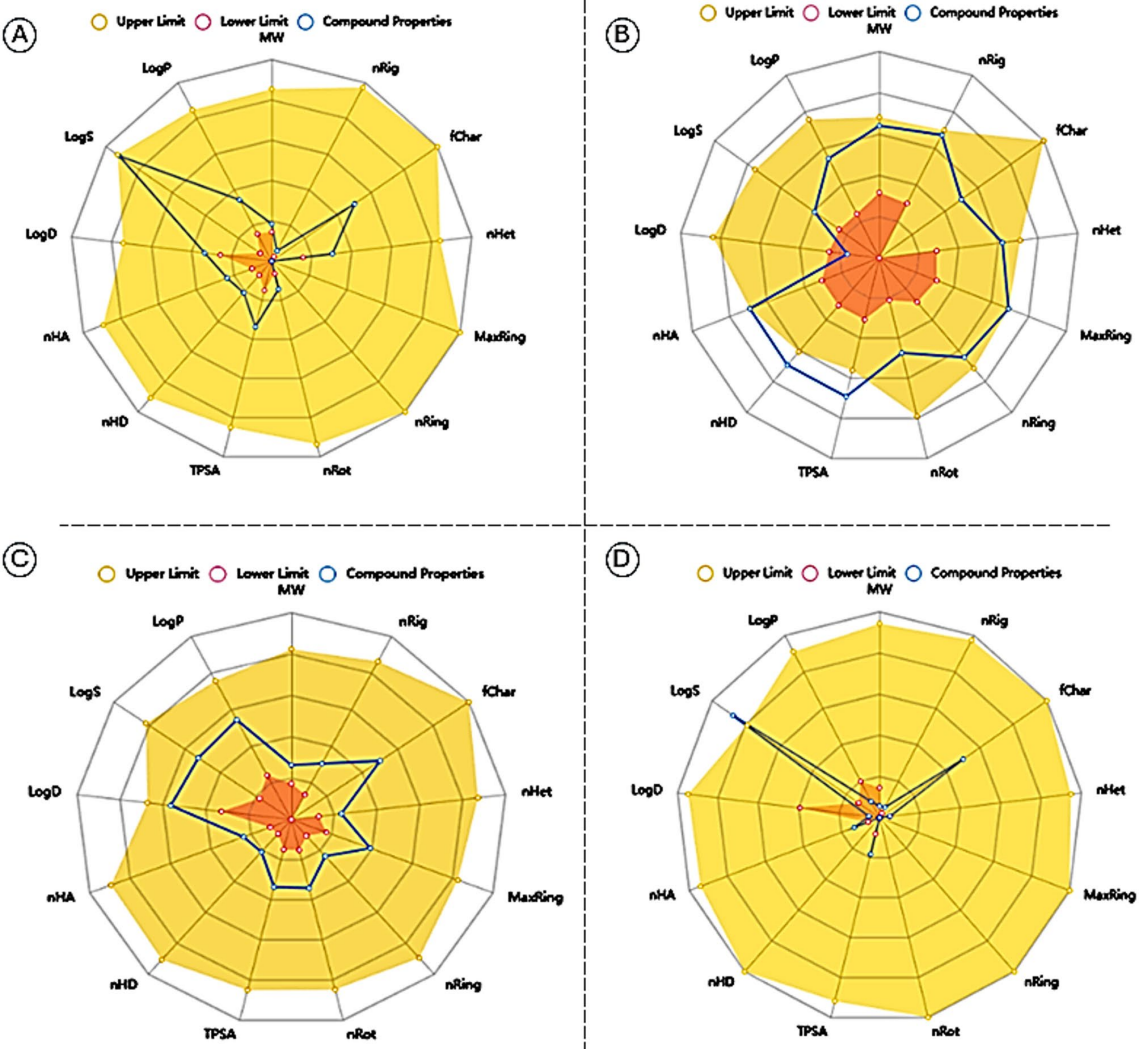
ADMET radar chart of ligands: (**A**) DCA, (**B**) Dox, (**C**) PLGA, and (**D**) PVA.

ADMET analysis revealed that Dox had higher hydrogen bonds acceptors (nHA) and donner (nHB) than DCA, PLGA, and PVA: (12, 2, 2, 1) for nHA and (9, 1, 1, 0) for nHB. DCA, PLGA, and PVA adhered to Lipinski, Pfizer, and GSK rules, while Dox was rejected. The pharmacokinetic profiles show Dox has highest clearance CL (9.566 mL/min/kg), half-life T1/2 (0.847 h), and PPB (91.29%). DCA shows moderate CL (6.035 mL/min/kg), longest T1/2 (0.895 h), and lower PPB (32.87%). PLGA and PVA have high CL with lower PPB (31.3 and 41.73%). The boiled egg construction (radar chart) revealed DCA had lower intestinal absorption (HIA = 0.505) but higher ideal drug properties with low toxicity to liver (H-HT = 0.049, DILI = 0.029) and heart (hERG = 0.003), compared to Dox with high absorptivity (HIA = 0.751) but higher toxic effects (H-HT = 0.275, DILI = 0.964, hERG = 0.019). PLGA and PVA showed moderate drug properties with high intestinal absorption and lowest toxicity. Dox exhibited highest carcinogenicity (0.776), stress response (SR-ARE = 0.813, SR-MMP = 0.959, SR-p53 = 0.988), and complex CYP3A4 metabolism with higher drug-drug interaction risk. DCA, PLGA, and PVA showed lower carcinogenic effect (0.029, 0.011, and 0.012), stress response, and favourable ADMET profile with low CYP-related interaction risk, as shown in (Table S1, Fig. 5A–D).

### DCA-PNPs reduced the % B.wt. change in EAC-bearing mice

**Fig. 6:**
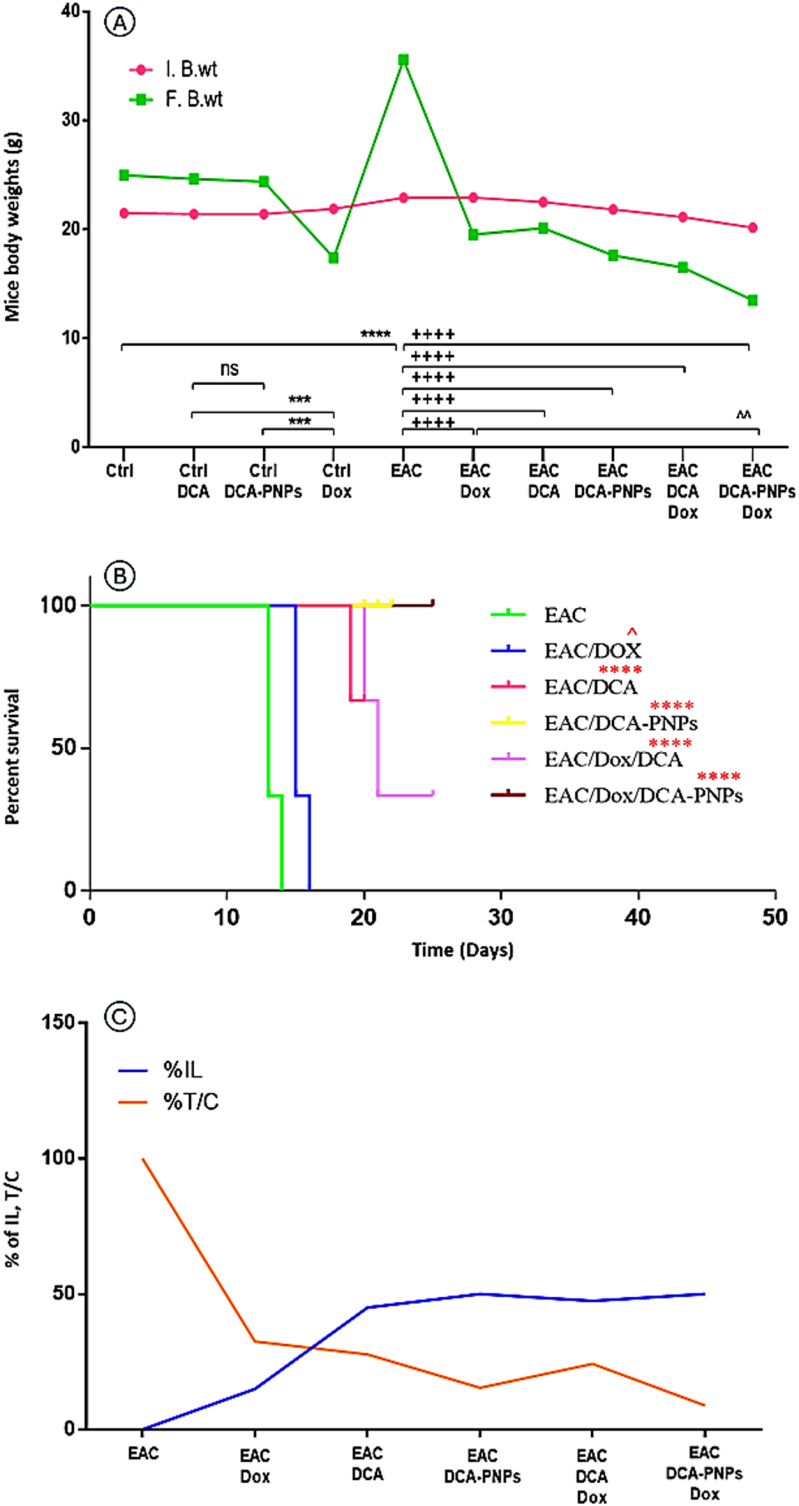
(**A**) Body weights at experiment start and end. (**B**) Mean survival duration in EAC bearing mice and all treated groups. (**C**) Life span (IL%) and tumor growth change (T/C%). Where (*p < 0.0001) is deemed significant to Ctrl group and (^*p* < 0.001, + *p* < 0.0001) denoting comparison to untreated EAC-bearing group.

As compared to the normal control group (Gp1), there was non-significant change in B. wt % in mice treated with DCA (Gp2) and DCA-PNPs (Gp3). However, normal mice which had treated with Dox (Gp4), showed a significant decrease in B.wt % by − 20.45%, (*p* < 0.0001). EAC-bearing mice (Gp5) showed a significant increase in B.wt % by + 55.21%, (*p* < 0.0001). As compared to Gp5, there was significant reduction in B.wt % in EAC mice which had treated with Dox (Gp6), DCA (Gp7), DCA-PNPs (Gp8), Dox/DCA (Gp9), and Dox/DCA-PNPs (Gp10) by − 14.58, − 10.5, − 19.25, − 21.18, − 33.05%, respectively (*p* < 0.0001) (Table S2 Fig. 6A).

### DCA-PNPs affect MST, ILS %, and T/C % in EAC-bearing mice

As compared to EAC untreated group (Gp5), there was significant increase in MST in EAC/Dox to EAC/Dox/DCA-PNPs (Gp6 to Gp10) represented as 15.3 ± 0.5, 19.3 ± 0.5, 20 ± 0.01, 19.6 ± 0.5, 20 ± 0.01 days, respectively (*p* < 0.0001) (Fig. 6B). As well, there was a significant increase in ILS % and a significant reduction in T/C % in EAC/Dox to EAC/Dox/DCA-PNPs (Gp6 to Gp10) (*p* < 0.0001) when compared to EAC untreated group (Gp5) (Fig. 6C).

### DCA-PNPs decreased the ascitic volume and tumor cell count

**Fig. 7:**
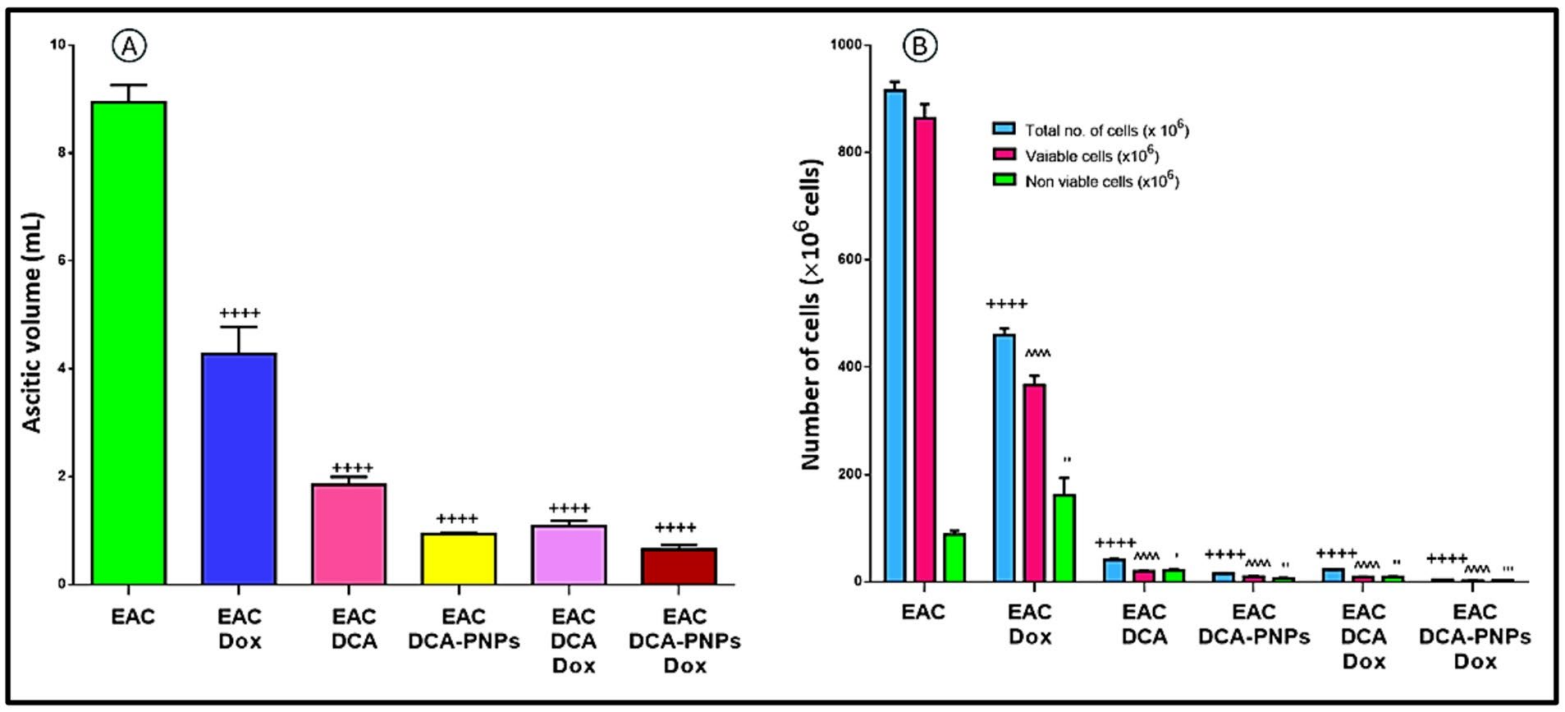
(**A**) Ascitic volume of EAC-bearing mice and all treated groups, (**B**) Number of viable, non-viable, and total cells in each EAC group. + *p*, compared to EAC-bearing mice group, total cell number, *^p* versus viable cells, and *'p* versus non-viable cells.

As compared to EAC untreated group (Gp5), there a significant reduction in ascitic volume in EAC/Dox to EAC/Dox/DCA-PNPs (Gp6 to Gp10) represented as 4.2 ± 0.5, 1.85 ± 0.15, 0.94 ± 0.02, 1.082 ± 0.09, 0.65 ± 0.09 mL, respectively (*p* < 0.0001) (Fig. S6; Fig. 7A). Also, there was a significant decrease in total EAC cell count in EAC/Dox to EAC/Dox/DCA-PNPs (Gp6 to Gp10) by −50.19, −95.53, −98.3, −97.5, and −99.54%, respectively (*p* < 0.0001) (Table S3; Fig. 7B).

### PDK1-4 expression decreased on cellular and mitochondrial levels by DCA-PNPs

**Fig. 8:**
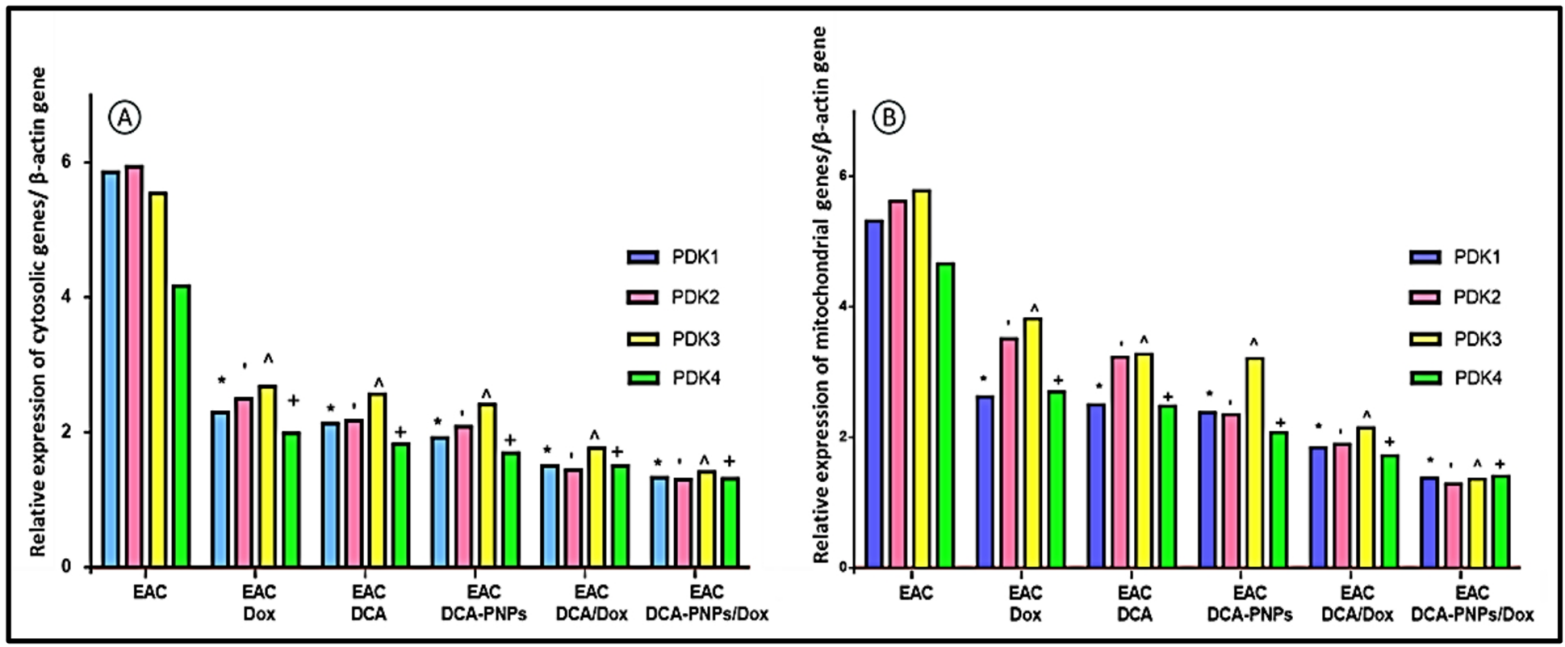
Relative expression of PDK1-4 against β actin housekeeping gene in EAC untreated and all treated groups (**A**) cytosol, and (**B**) mitochondria. (^*,‘,^,+^*p* < 0.0001) which significant to PDK1,2,3,4 respectively for all treated groups vs. EAC-bearing group.

As compared to EAC untreated group (Gp5), qRT-PCR revealed significant downregulation (*p* < 0.0001) of cellular and mitochondrial PDK1, PDK2, PDK3, and PDK4 genes expression in EAC/Dox to EAC/Dox/DCA-PNPs (Gp6 to Gp10). Interestingly, the most downregulated folds were in Dox/DCA-PNPs (Gp10) treated group by folds 1.32 ± 0.53 for PDK1, 1.30 ± 0.37 for PDK2, 1.41 ± 0.42 for PDK3, and 1.31 ± 0.1 for PDK4 in cells. As well in mitochondria, fold changes significantly increased by 1.3 ± 0.07 for PDK1, 1.28 ± 0.03 for PDK2, 1.35 ± 0.03 for PDK3, and 1.4 ± 0.19 for PDK4. Which indicated effectively reduced reliance on glycolytic pathway, promoted cancer cell death, and significantly restored of mitochondrial function (Fig. 8, Fig. S1, S2, S3, S4, S5; Table S4).

### DCA-PNPs induced apoptotic profile in EAC cells

**Fig. 9:**
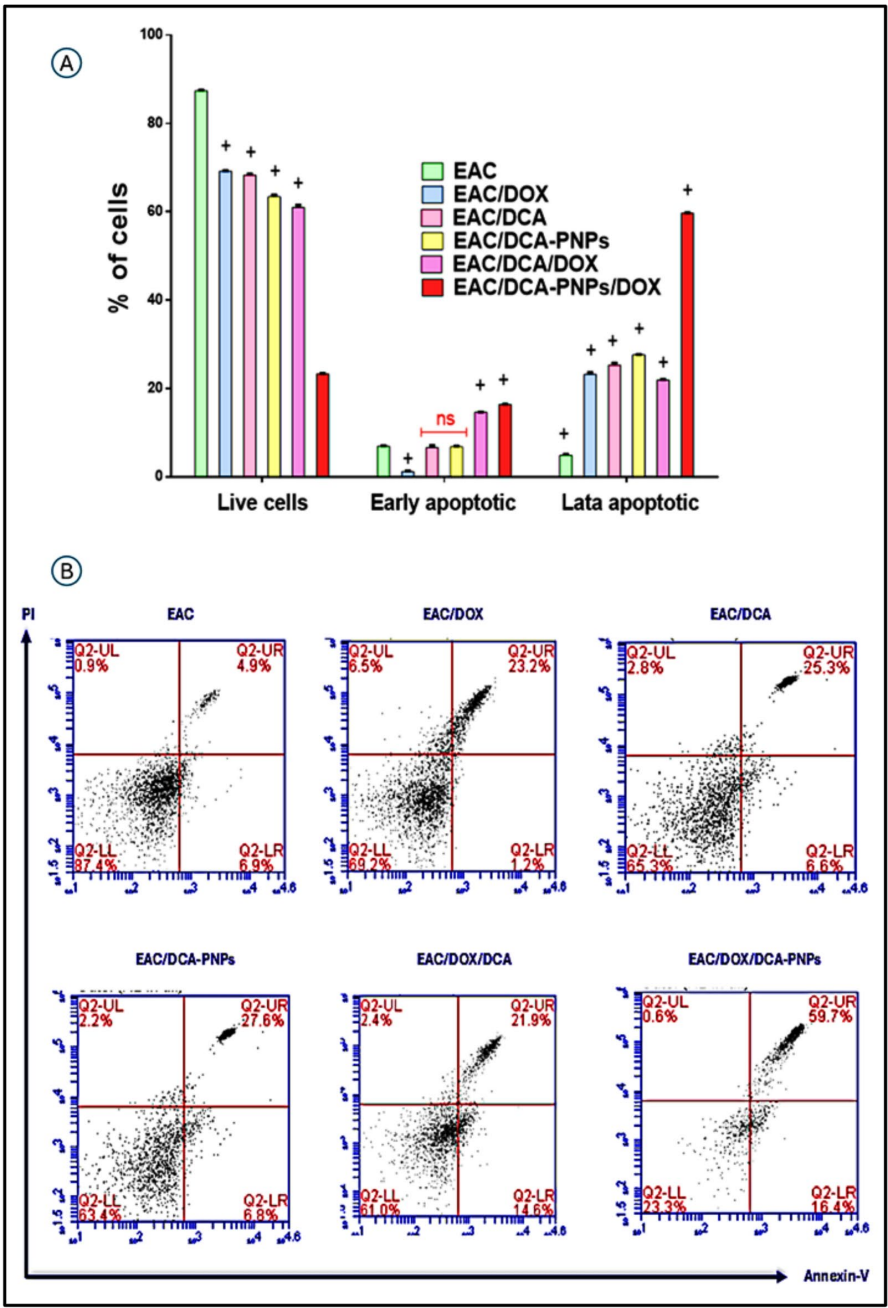
(**A**) Annexin V cell percentage and (**B**) dot plot of EAC-bearing mice group and all treated groups. Results were expressed as mean ± SE n = 4. (*p* value < 0.0001) is significantly expressed, where ^+^ significant in all treated groups vs. EAC-bearing mice.

Live cells, early apoptotic, and late apoptotic percentage of EAC cells was analyzed using flow cytometry after treatment with Dox (Gp6), DCA (Gp7), DCA-PNPs (Gp8), Dox/DCA (Gp9), Dox/DCA-PNPs (Gp10) and the phenotypic distributions of live cells (Annexin^−^PI^−^), early apoptotic (Annexin^+^PI^−^), and late apoptotic (Annexin^+^PI^+^) are displayed in Fig. 9A and B. The results showed a significant decrease in live cells % in EAC/Dox to EAC/Dox/DCA-PNPs (Gp6 to Gp10) represented as 69.2, 65.3, 63.4, 61, and 23.3%, respectively (*p* < 0.0001) as compared to EAC untreated group (Gp5). As compared to EAC untreated group (Gp5), early apoptotic % was significantly decreased in EAC/Dox (Gp6) to 1.2%, (*p* < 0.0001) and significantly increased in EAC/Dox/DCA (Gp9) and EAC/Dox/DCA-PNPs (Gp10) as 14.6 and 16.4%, respectively (*p* < 0.0001); but there was non-significant change in EAC/DCA (Gp7) and EAC/DCA-PNPs (Gp8). As compared to EAC untreated group (Gp5), late apoptotic % was significantly increased in EAC/Dox to EAC/Dox/DCA-PNPs (Gp6 to Gp10) represented as (23.2, 25.3, 27.6, 21.9, and 59.7%). According to that, EAC/Dox/DCA-PNPs (Gp10) was the most effected group, demonstrating a synergistic effect in inducing cancer cell death.

### DCA-PNPs arrested G0/G1 phase cell cycle arrest in EAC cells

**Fig. 10:**
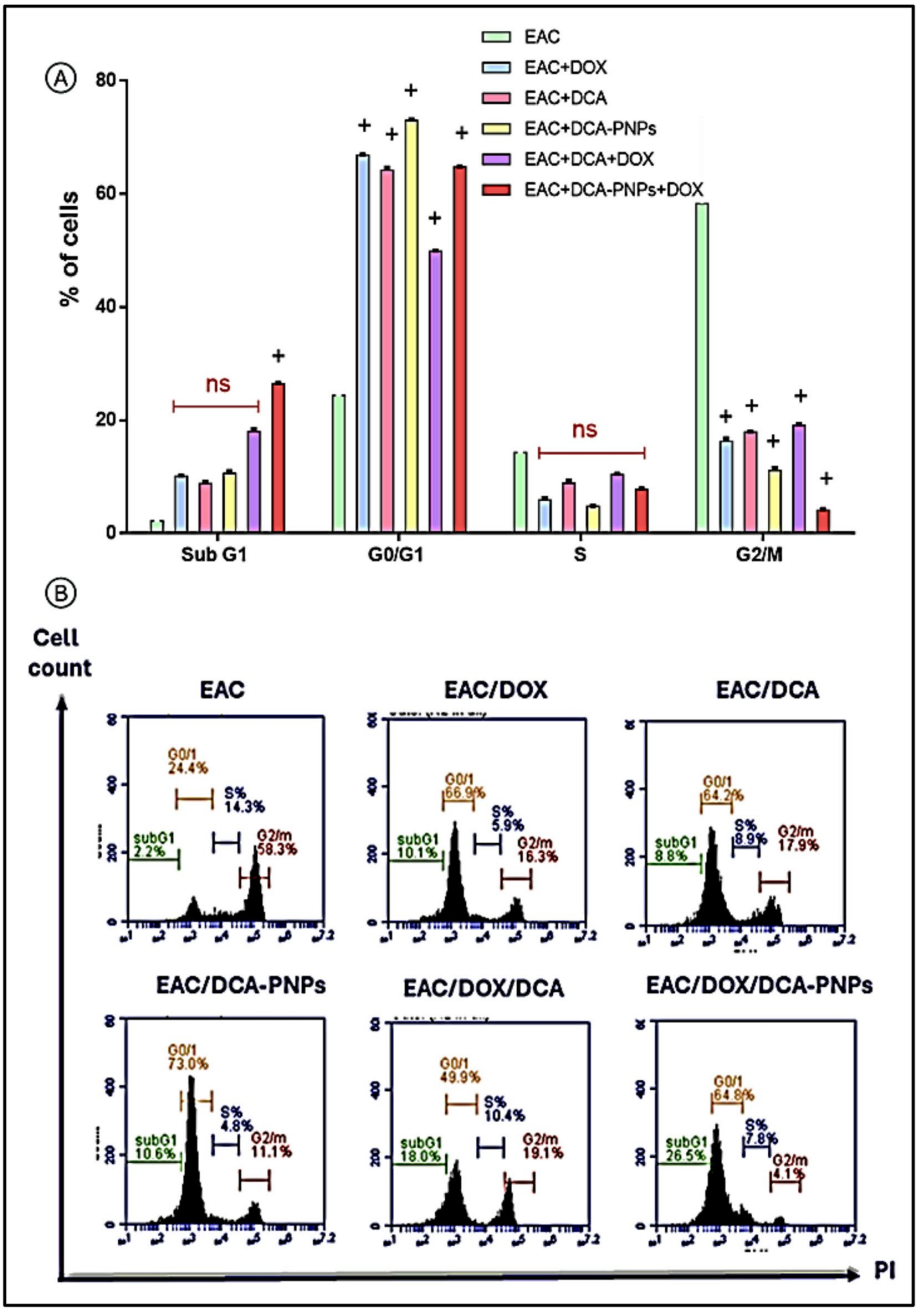
(**A**) Cell cycle arrest phases percentage and (**B**) chromatogram of all treated groups and EAC-bearing mice group. Results were expressed as mean ± SE n = 4. (*p* value < 0.0001) is significantly expressed, where ^+^ significant in all treated groups vs. EAC-bearing mice.

Cell cycle analysis showed that EAC/Dox to EAC/Dox/DCA-PNPs (Gp6 to Gp10) were significantly increased by this order [(G0/G1: 66.9%, S: 5.9%, G2/M: 16.3%, sub G1: 10.1% for Gp6) < (G0/G1: 64.2%, S: 8.9%, G2/M: 17.9%, sub G1: 8.8% for Gp7) < (G0/G1: 73%, S: 4.8%, G2/M: 11.1%, sub G1: 10.6% for Gp8) < (G0/G1: 49.9%, S: 10.4%, G2/M: 19.1%, sub G1: 18% for Gp9) < (G0/G1: 64.8%, S: 7.8%, G2/M: 4.1%, sub G1: 26.5% for Gp10)] *(p* < 0.0001), in the cell cycle arrest specially at the G0/G1 phase when compared to the G2/M phase in EAC untreated group (Gp5), which showed high tumor cells proliferation (G0/G1: 24.4%, S: 14.3%, G2/M: 58.3%, sub G1: 2.2%). Based on that, the highest G0/G1 arrest was in EAC/DCA-PNPs (Gp8) and the highest sub G1 was in EAC/Dox/DCA-PNPs (Gp10), which indicated effective halting of cell cycle progression, preventing cancer cell proliferation by DCA-PNPs alone and in combinatory treatment (Fig. 10 A, B).

### DCA-PNPs improved liver and kidney pathological architecture

**Fig. 11:**
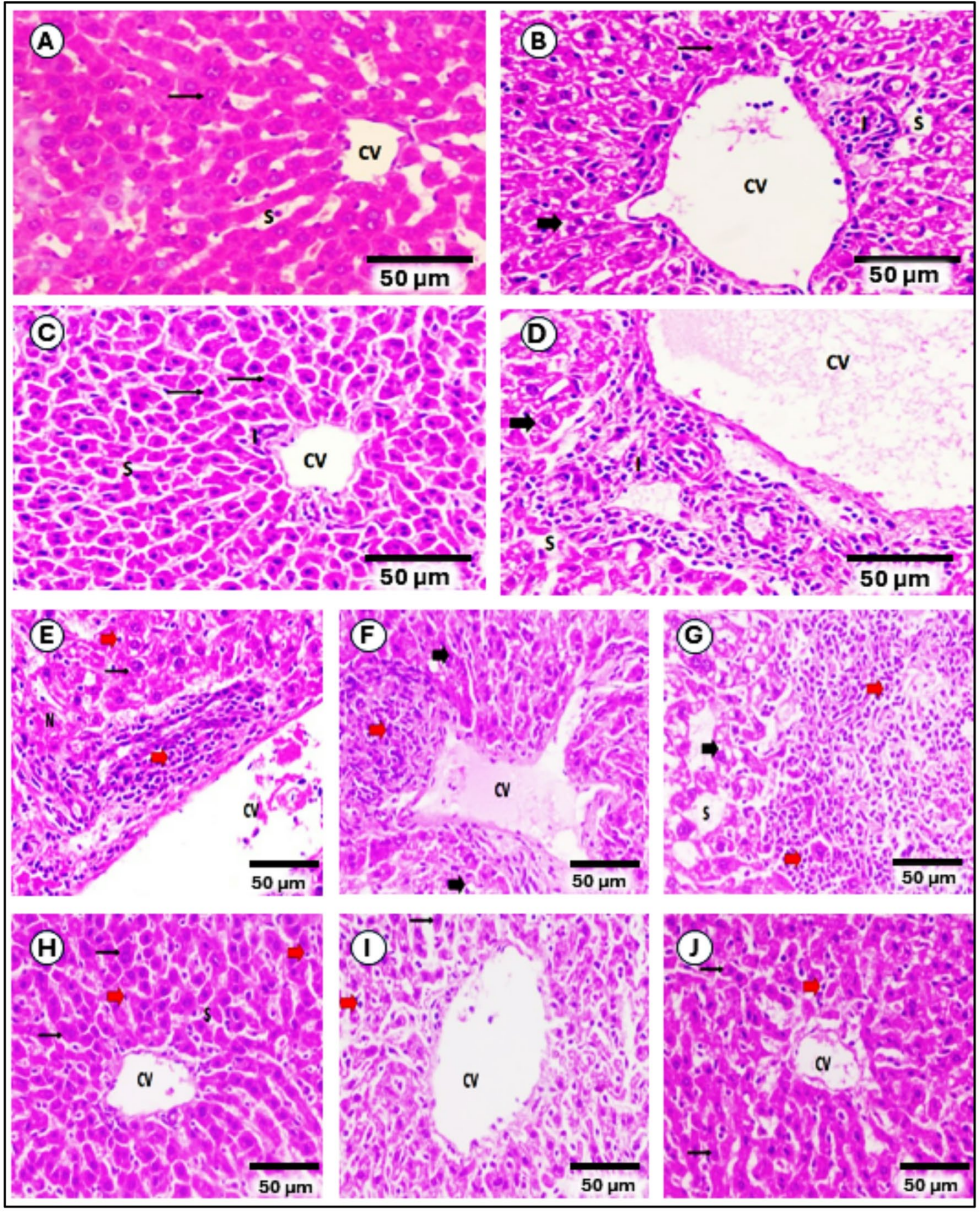
Histopathology of liver sections in all groups: Normal group (**A**); Ctrl/DCA group (**B**); Ctrl/DCA-PNPs group (**C**); Ctrl/DOX group (**D**); EAC group (**E**); EAC/DOX group (**F**); EAC + DCA group (**G**); EAC/DCA-PNPs group (**H**); EAC/DCA/DOX group (**I**); EAC/DCA-PNPs/DOX group (**J**). Where central vein (CV), blood sinusoids (S), normal binucleated hepatocytes with moderately acidophilic cytoplasm (thin arrow), rarified cytoplasm and vacuolar degeneration of hepatocyte (thick arrow), inflammatory infiltrate (I), focal necrosis (N), focal and diffuse tumor cells (red arrows), (H&E Max. 400).

Normal group (Gp1) liver architecture showed well-organized hepatocyte cords, sinusoids, central veins, and portal triads (Fig. 11A). Similar to it Ctrl/DCA (Gp2), and Ctrl/DCA-PNPs (Gp3) showed mild changes suggesting minor injury, and nearly normal architecture, indicating minimal toxicity (Fig. 11B, C). But Ctrl/Dox (Gp4) showed marked alterations indicating significant injury (Fig. 11D). EAC-bearing group (Gp5) showed severe damage with tumor infiltration by presence of undifferentiated, pleomorphic tumor cells that infiltrate and disrupt the liver architecture (Fig. 11E). As compared to EAC-bearing group (Gp5), there was no substantial alteration in liver architecture was observed within EAC/Dox (Gp6), (Fig. 11F). In contrast, there was a significant improvement in EAC/DCA (Gp7), EAC/DCA-PNPs (Gp8), EAC/Dox/DCA (Gp9), and EAC/Dox/DCA-PNPs (Gp10), indicated as improved architecture suggesting partial recovery, effective reversal of EAC-induced damage, dilated central veins, few normal hepatocytes, focal tumor cells showed for, and improved architecture, respectively. EAC/Dox/DCA-PNPs (Gp10) demonstrated the most effective recovery among treated groups, as it had more similarity to normal group (Fig. 11G–J).

**Fig. 12:**
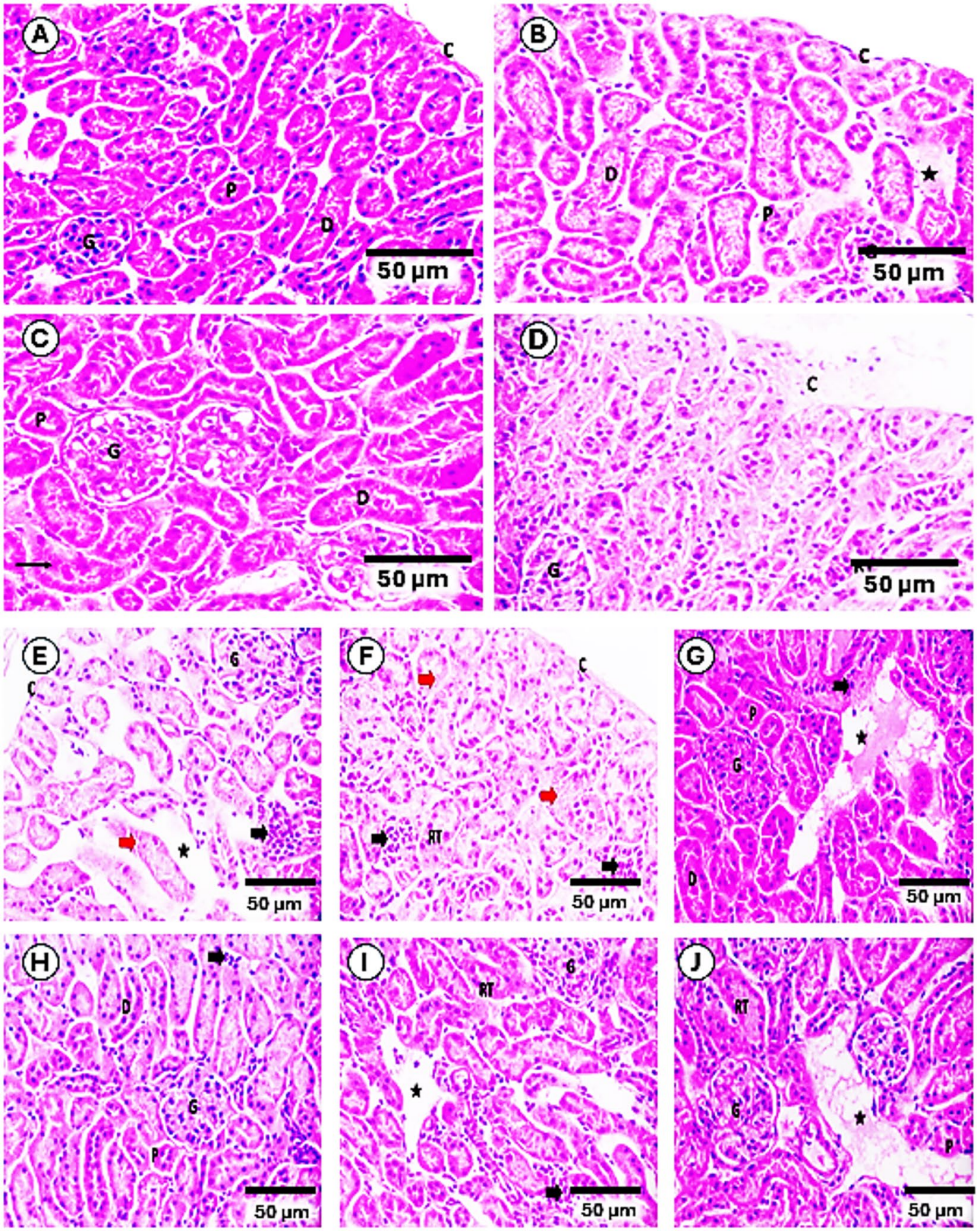
Histopathology of kidney sections in all groups: Normal group (**A**); Ctrl/DCA group (**B**); Ctrl/DCA-PNPs group (**C**); Ctrl/DOX group (**D**); EAC group (**E**); EAC/DOX group (**F**); EAC + DCA group (**G**); EAC/DCA-PNPs group (**H**); EAC/DCA/DOX group (**I**); EAC/DCA-PNPs/DOX group (**J**). Where intact capsule (C), glomeruli (G), normal proximal convoluted tubules (P), distal convoluted tubules(D), interstitial tissue (*), focal disruption (thin arrow) or disrupted histological architecture of renal tubule (RT), tumor cells (thick arrow), necrosis of renal tubules (red arrow), and interstitial tissue (*), (H&E Max. 400).

Normal group (Gp1) kidney architecture showed well-organized glomeruli, tubules, and minimal interstitium (Fig. 12A). As compared to it, Ctrl/DCA (Gp2), Ctrl/DCA-PNPs (Gp3), Ctrl/Dox (Gp4) showed mild renal injury, nearly normal kidneys with focal tubule disruption, and significant renal damage, respectively (Fig. 12B–D). EAC-bearing group (Gp5) showed severe cortical injury with tumor infiltration by presence of pleomorphic tumor cells, nuclear atypia, stromal changes, and vascular invasion, indicative of malignant transformation (Fig. 12E). As compared to EAC-bearing group (Gp5), there was intact capsules but focal tumor cells and necrotic areas in EAC/Dox (Gp6), (Fig. 12F). While there was a significant improvement in all other treated groups, which showed improved architecture with fewer tumor cells for EAC/DCA (Gp7), marked improvement with few remaining tumor cells for EAC/DCA-PNPs (Gp8), normal glomeruli, focal tumor cells, and distorted tubules for EAC/Dox/DCA (Gp9), and improved architecture for EAC/Dox/DCA-PNPs (Gp10) as the most similar to normal, indicating the most effective recovery among treated Groups (Fig. 12G–J).

**Fig. 13:**
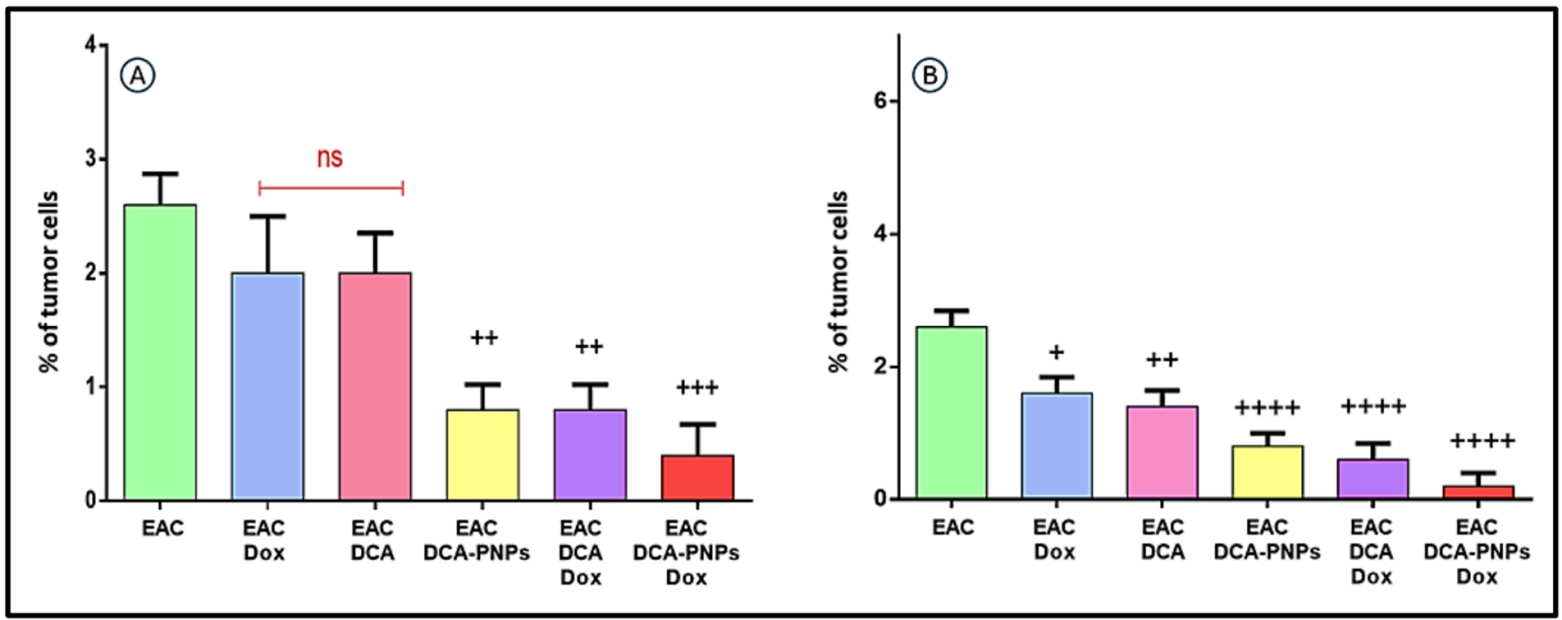
(**A**) Histopathological scoring of liver tissue, (**B**) and histopathological scoring renal cortex tissue in all treated groups vs EAC untreated group. Results were expressed as mean ± SE n = 4. (+ *p* value < 0.0001) vs. EAC untreated group.

Histopathological scoring in liver and renal cortical injury confirmed observations. Graded on (hepatocyte degeneration, necrosis, inflammatory infiltration, tumor cell presence) in liver, (tubular necrosis, tumor cell infiltration, and interstitial widening) in kidney. EAC untreated group (Gp5) showed highest score (grade 3), indicating severe damage. Treated groups showed progressively lower scores, with EAC/DCA-PNPs/DOX group (Gp10) exhibiting the most significant improvement, highlighting potential of combined therapy in mitigating tissue injury (Table S4; Fig. 13A, B).

## Discussion

Breast cancer is a significant global health threat, with increasing incidence and mortality rates^1^. The Warburg effect, characterized by increased glycolysis and decreased oxidative phosphorylation, promotes cancer cell progression^3^. This study aimed to evaluate the binding strength between DCA and PDKs through molecular docking and elucidate the synergistic impact of DCA-PNPs/Dox combinatorial treatment on suppressing PDKs in EAC cells. Dox remains the first-line chemotherapeutic agent for breast cancer^15^; however, its therapeutic efficacy is often limited by drug resistance and dose-dependent toxicity^16^. Therefore, combining Dox with other agents such as DCA represents a promising strategy to enhance anticancer efficacy, overcome metabolic adaptation, and minimize adverse effects. In the current study DCA-PNPs/Dox combination demonstrates remarkable efficacy in inhibiting tumour growth of the EAC model, a mammary adenocarcinoma model commonly used to investigate breast cancer, reducing oxidative stress, and restoring mitochondrial function through targeted inhibition of PDK enzymes, which are critical in cancer metabolism. Study supposed that DCA-PNPs formulation could enhance Dox efficacy against tumor proliferation and target cancer cells perfectly without affecting normal cells, which is consistent with previous study^25^.

Nanoparticles (NPs) characterizations as high EE% and DL% values indicate efficient drug entrapment, suggesting optimal utilization of the drug and nanocarrier materials^26^. Such high encapsulation is crucial for maximizing therapeutic outcomes, as it allows sustained drug release and reduces systemic side effects. Our findings are consistent with those of Mostafa et al. (2023), who also reported enhanced encapsulation efficiency using PLGA-based nanoparticles. Interestingly, the slightly higher EE% achieved in our study may be attributed to the stabilizing effect of PVA, which improves nanoparticle formation and drug entrapment, a parameter not optimized in previous work^27^. TEM and SEM imaging techniques visualize NP morphology, size, and distribution, that uniform particle size and shape confirm successful nanoparticle formation and provide direct evidence of loading^28^. This is in line with Todaro et al. (2022), but unlike their report, our particles exhibited a narrower size distribution, likely due to the optimized emulsification parameters applied in our formulation protocol. Spectroscopic characterization supported these structural observations. UV analysis revealed a distinct red shift in the absorption peak of DCA after encapsulation, confirming successful drug incorporation and possible interactions with PLGA. This shift was more pronounced than that reported by Gharbavi et al.^29^, suggesting stronger drug–polymer interactions in our system, potentially influencing release kinetics. FTIR spectra reveal the chemical composition and interactions within the NP system, that presence of characteristic peaks from both the drug and nanocarrier materials, along with potential peak shifts or new peaks, indicates successful drug loading and possible drug-nanocarrier interactions^30^. That in this study, FTIR of DCA-PNPs showed a peak that shifted in between DCA and PLGA due to the absence of DCA’s characteristic bands in DCA-PNPs but in the same functional groups range as the peak at 1683 cm^−1^, which congruent with prior research^31^. That indicates effective encapsulation within the NP core and suggests DCA compatibility with PLGA, the main excipient in NP fabrication. Moreover, XRD patterns indicated a partial transition of DCA from a crystalline to a more amorphous state upon encapsulation, which can enhance drug solubility and release a phenomenon previously described for similar nanocarriers^32^. Finally, zeta potential is a critical parameter in NP loading and stability, that measures the electrical potential difference between the bulk of a conducting medium and the stationary layer of fluid attached to the dispersed particle^33^. In current study, DCA-PNPs broad and low intense band in between sharp DCA and amorphous PLGA bands suggesting its crystallization and encapsulation, which agreed with previous study by Qi et al. (2021) findings which support that slightly negative zeta potential values observed reflect the influence of PLGA’s surface charge and predict good colloidal stability^34^. Where the slight broadness of the zeta potential peaks reflects the natural distribution of surface charge in polymeric nanoparticles and does not compromise their stability, as supported by the negative ζ-potential values measured.

According to these physicochemical characterization of DCA-PNPs (TEM, SEM, FTIR, UV–Vis, Zeta potential and XRD) indicates that DCA was successfully encapsulated and intact without substantial alteration of its molecular structure. Collectively, these data support the preservation of DCA’s structural integrity within the PLGA matrix during formulation.

Molecular docking analysis provided mechanistic insights into the interactions of DCA, Dox, PLGA, and PVA with pyruvate dehydrogenase kinases (PDKs), which play a pivotal role in cancer metabolic reprogramming^35^. DCA exhibited the strongest binding affinity among all tested ligands, occupying the catalytic pocket of PDK1 and PDK4 with optimal orientation and multiple hydrogen bonds, suggesting a stable and inhibitory complex formation. This supports its well-established role as a PDK inhibitor and aligns with previous reports by She et al. (2023), who demonstrated that dichloroacetate derivatives effectively disrupt PDK activity, thereby restoring mitochondrial oxidative metabolism^36^. Notably, the higher binding score obtained in our study compared to theirs may be attributed to the structural compatibility of DCA within the PLGA matrix, potentially enhancing local concentration at the target site. PDKs, forming multiple hydrogen bonds and hydrophobic interactions within the binding pocket. This suggests a complementary inhibitory mechanism that could synergize with DCA to potentiate metabolic reprogramming, consistent with the cooperative effect reported by Chen et al.^37^. The polymer PLGA displayed moderate affinity toward the enzyme surface, adopting a conformation that supports nanoparticle–protein interactions and potentially influencing drug release kinetics and targeting. Meanwhile, PVA’s flexible backbone enabled multiple contact points with PDKs, indicating a possible role in stabilizing the drug–enzyme complex, a property rarely reported for excipients and warranting further investigation.

ADMET (Absorption, Distribution, Metabolism, Excretion, and Toxicity) profiling provided additional evidence supporting the suitability of these components for therapeutic application.^38^. DCA displayed favorable pharmacokinetic characteristics, including high oral bioavailability, acceptable metabolic stability, low predicted toxicity, and compliance with Lipinski’s rule of five. These findings are consistent with previous in silico analyses^39^ and support its potential for systemic administration. In contrast, Dox exhibited limited oral absorption and notable cardiotoxicity risks (e.g., hERG inhibition and DILI potential), findings in agreement with Prasad et al.^40^, underscoring the importance of nanoparticle-based delivery to mitigate these limitations. The polymers demonstrated complementary ADMET properties: PLGA showed excellent biodegradability, biocompatibility, and favourable elimination pathways^41^, despite low oral bioavailability, exhibited an outstanding safety profile with minimal systemic absorption and renal excretion^42^. Collectively, these findings indicate that the combination of DCA and Dox within a PLGA–PVA nanocarrier can optimize therapeutic efficacy by enhancing PDK inhibition and minimizing systemic toxicity.

Tumor progression in the EAC model is typically characterized by progressive increases in body weight, ascitic fluid volume, and viable tumor cell count, accompanied by decreases in mean survival time (MST) and life span percentage^43,44^. Consistent with this pattern, untreated tumor-bearing mice in the present study exhibited substantial ascitic accumulation and rapid tumor cell proliferation, highlighting the aggressive nature of the model. In contrast, treatment with DCA-PNPs/Dox markedly attenuated these hallmarks of tumor progression, as evidenced by significant reductions in body weight gain, ascitic fluid volume, and viable tumor cell count, coupled with significant prolongation of MST and LS%. The observed decrease in T/C% further underscores the potent antitumor efficacy of the formulation, as lower T/C% ratios correlate with greater tumor growth inhibition^10,45^. These results are in strong agreement with previous reports demonstrating that nanoparticle-based delivery systems can effectively reduce tumor burden and mitigate cancer-associated weight gain^46,47^. Our findings also corroborate studies highlighting the synergistic therapeutic benefits of combining DCA and doxorubicin, which can enhance tumor suppression and survival outcomes in various cancer models^5,7,16,48^. Notably, the magnitude of ascitic volume reduction observed in our study was greater than that reported by Rahmani et al.^16^, possibly due to improved biodistribution and cellular uptake conferred by the PLGA–PVA nanocarrier. This enhanced delivery likely results in higher intratumoral drug concentrations and more effective metabolic reprogramming via PDK inhibition, thereby suppressing tumor cell proliferation. Because EAC is an ascitic tumor model, the extent of tumor burden is directly reflected by the accumulation of malignant ascitic fluid rather than by a solid mass^10^. Therefore, the substantial decrease in ascitic fluid volume following treatment indicates a profound reduction in tumor load. This conclusion is visually supported by representative images (Fig. S6), which show markedly less ascitic fluid in treated mice. Together with the quantitative reductions in tumor cell count, these observations confirm that DCA-PNPs/Dox therapy significantly delays tumor progression and improves survival, reinforcing the therapeutic potential of this combinatorial nanoparticle system against rapidly proliferating ascitic tumors.

A key finding of our study was the significant downregulation of all four PDK isoforms (PDK1–4) in both cytosolic and mitochondrial fractions of EAC cells following DCA/Dox treatment. Notably, PDK1 and PDK2 exhibited the greatest sensitivity, which is consistent with their central regulatory role in PDC activity and aligns with prior studies demonstrating that these isoforms are preferentially inhibited by DCA^49,50^. Interestingly, although PDK3 showed relatively lower downregulation—consistent with its tissue-specific expression and resistance to DCA inhibition^51,52^—this partial inhibition still supports metabolic reprogramming. This suggests that even modest suppression of PDK3 contributes to shifting cancer metabolism away from glycolysis. The marked decrease in PDK4 further underscores the treatment’s impact on metabolic adaptability, which is particularly relevant because PDK4 upregulation has been linked to enhanced metastasis and resistance in breast cancer^53,54^. These findings partially confirm study docking findings for Dox and DCA, where PDK1 and PDK3 were the most inhibited. While was completely agreed with previous studies suggested that PDK1 and PDK2 is the most effected by DCA inhibition, while Dox inhibited PDK1, PDK2, and PDK4 more than PDK 3. These complementary computational and experimental findings confirm that the therapeutic response of PDK isoforms is not uniform but isoform-specific, with PDK1 and PDK4 emerging as the most responsive targets. This synergy between docking predictions and gene expression results provides a mechanistic explanation for the reactivation of PDC and the metabolic shift from glycolysis to oxidative phosphorylation observed in treated EAC cells. This downregulation promotes the reactivation of the PDC and shifting cancer cells from glycolysis to oxidative phosphorylation. This metabolic reprogramming is essential for diminishing cancer cell viability and proliferation as in previous studies explained PDKs expression role in details to control cancer metabolism^3,20^.

Several anticancer compounds demonstrate their growth inhibitory effect either by induction of apoptosis or by arresting the cell cycle at a specific checkpoint of cell cycle or a collective effect of both apoptosis and cycle arrest^55^. Our findings revealed that treatment with the DCA-PNPs/Dox combination induced the most pronounced apoptotic response in EAC cells, significantly increasing both early and late apoptotic populations while markedly reducing viable tumor cells. The apoptotic effect observed with DCA-PNPs alone was also substantial and exceeded that of DCA or Dox monotherapy, underscoring the contribution of nanoparticle-mediated delivery to therapeutic potency. These observations support the concept that nanoparticle formulations can enhance intracellular drug accumulation and prolong retention, thereby amplifying pro-apoptotic signalling^56^. They are also consistent with previous work demonstrating that DCA triggers apoptosis in EAC cells by reprogramming cellular metabolism and disrupting mitochondrial function^57^, but our data extend these findings by showing that co-delivery with Dox further intensifies this effect.

The cell cycle analysis provided complementary evidence of treatment efficacy. Flow cytometric profiling demonstrated distinct arrest patterns among treatment groups, with the DCA-PNPs/Dox group inducing a pronounced accumulation of cells in the sub-G1 phase a hallmark of apoptosis-associated DNA fragmentation^56,58^. DCA-PNPs alone predominantly arrested the cell cycle at the G0/G1 checkpoint, suggesting that part of their antitumor activity involves preventing cell cycle progression prior to DNA synthesis. This G0/G1 arrest may initially represent a cytostatic response that transitions into apoptosis with prolonged exposure. Such dual-phase responses have been described for several nanomaterial-based therapies, which first impose cell cycle blockade and subsequently activate apoptotic pathways^18,59^ Interestingly, the magnitude of G0/G1 arrest observed with DCA-PNPs exceeded that induced by Dox alone, indicating that the nanoparticle formulation exerts a stronger cytostatic pressure on tumor cells. This may be related to enhanced cellular uptake and sustained metabolic disruption mediated by DCA, which sensitizes cells to apoptosis^60^. These findings align with previous studies showing that nanocarriers loaded with chemotherapeutics significantly potentiate both apoptosis and cell cycle arrest compared to free drug treatments^55,61^. Moreover, our results contribute an insight by demonstrating that combining metabolic modulation (via DCA) with a DNA-damaging agent (Dox) can synergistically drive EAC cells toward irreversible growth arrest and programmed cell death a therapeutic approach that could be explored further in solid tumor models.

With regard to histological investigations, which provided crucial evidence supporting the biochemical and molecular findings of this study. As expected, liver and kidney tissues from the untreated control group (Gp1) exhibited normal histoarchitecture, confirming baseline physiological status. Minor structural changes observed in Ctrl/DCA (Gp2) and Ctrl/DCA-PNPs (Gp3) groups suggest that DCA and its nanoparticle formulation exert minimal intrinsic toxicity on healthy tissues, which is consistent with their known safety profile^88^. In contrast, the Ctrl/Dox (Gp4) group displayed marked hepatocellular and renal alterations, reflecting the well-documented organ toxicity associated with doxorubicin, primarily mediated through oxidative stress and mitochondrial dysfunction^62^. EAC untreated (Gp5) exhibited severe hepatic and renal damage, including disrupted tissue organization and tumor infiltration, in agreement with previous studies reporting extensive organ injury associated with tumor progression^63,64^. Treatment with Dox alone (Gp6) did not substantially reverse these alterations in hepatic tissue, and only partial structural recovery was observed in renal tissue, indicating the limited therapeutic efficacy of Dox monotherapy in mitigating EAC-induced organ injury. In contrast, groups treated with DCA, DCA-PNPs, or their combinations (Gp7–Gp10) demonstrated clear histological improvements, with progressive tissue restoration observed across groups. Notably, the DCA-PNPs/Dox combination (Gp10) achieved near-normal architecture in both liver and kidney, highlighting the synergistic therapeutic potential of this treatment.

Histopathological scoring further corroborated these observations: the highest injury scores were recorded in the EAC-untreated group (Gp5), whereas progressively lower scores were observed with treatment, with the lowest score achieved in the DCA-PNPs/Dox group. These results reinforce the conclusion that combining DCA-PNPs with Dox enhances therapeutic efficacy while reducing systemic toxicity, aligning with reports showing that metabolic modulation by DCA can mitigate chemotherapy-induced organ damage^65–67^. Importantly, the study’s findings have broader implications beyond the EAC model. Although EAC is an ascitic tumor, it originates from a spontaneous mammary adenocarcinoma and is widely used as a surrogate for breast cancer. The observed inhibition of PDK activity, metabolic reprogramming toward oxidative phosphorylation, and significant reductions in tumor burden and tissue damage suggest that the DCA-PNPs/Dox strategy could be a promising therapeutic approach for breast cancer. These histopathological outcomes support a mechanistic model in which metabolic targeting (via PDK inhibition) not only suppresses tumor growth but also alleviates secondary organ injury associated with both tumor progression and chemotherapy, providing a dual therapeutic benefit^68^.

## Materials and methods

### Chemicals and drugs

Sodium dichloroacetate (DCA) (≥ 98%; Cat. no. 347795), poly D, L-lactic-co-glycolide (PLGA) (L: D 50:50, Mw 45,000; Cat. no. 805726), polyvinyl alcohol (PVA) (80–90% hydrolyzed, Mw 30,000–70,000, Cat. no. P8136), Doxorubicin hydrochloride (Dox) (98.0–102.0% HPLC, Mw 579.98, Cat. no. D1515), phosphate buffer saline (PBS) (pH 7.4, Cat. no. P4474), and acetone (≥ 99.5%, Cat. no. 179124) were purchased from Sigma-Aldrich, (Germany). RNA extraction ('Cat no. # K0731), and Reverse transcription (Cat. no. EP0451) kits purchased from Thermo Scientific, (Fermentas). PDK1-4 and housekeeping β-actin genes’ primers were from Biotech. Company, (Egypt). All other chemicals used were high grades.

### Synthesis of DCA-PNPs

Dichloroacetate nanoparticles were prepared via single emulsion solvent vaporization using acetone as an organic diluent for PLGA and PVA that acted as stabilizer. PVA was dissolved in distilled water (2.5%) by magnetic stirring at 2000 rpm for 1 h at 45 °C. PLGA and DCA were dissolved in acetone (1%) employing a magnetic stirrer at 1000 rpm for 5 min in ambient temperature. The organic solution was added to the PVA surfactant solution during high-speed centrifugation for 5 min in an ice bath. The solution underwent continuous magnetic swirling to promote the evaporation of the organic layer. DCA-PNPs were synthesised by stirring the mixture at 2000 rpm for 6 h in ambient temperature. DCA-PNPs were separated via high-speed centrifugation at 15,000 rpm for 30 min at 4 °C. The pellet was resuspended in 10 mL of distilled water and underwent lyophilization for 24 h to get DCA-PNP powder. Protocols were evaluated based on^69^ with some adjustments incuding adjusting tempreture degree, solution concentrations, and minimising evaporation time.

### Characterisation of DCA-Polymeric nanoparticles

#### Encapsulation efficiency and drug-loading capacity

To determine the encapsulation efficiency (%EE) and drug loading capacity (%DL) of DCA-PNPs, the suspension was subjected to centrifugation at 15,000 revolutions per minute for 15 min at 4 °C. The supernatant containing unencapsulated DCA was collected. A calibration curve was constructed by plotting medication concentrations against absorbance values obtained by HPLC–UV analysis. The concentration of free DCA in the supernatant was determined by measuring its absorbance at 265 nm (λmax) and comparing it to the calibration curve^69^. The total amount of DCA initially added was also quantified. The result was calculated as seen in Eqs. (1) and (2).

1 $$\% {\mathrm{EE}} = \left[ {{\text{Amount of DCA precipitation NPs }}\left( {{\mathrm{mg}}} \right)/{\text{ Initial amount of DCA used }}\left( {{\mathrm{mg}}} \right)} \right] \times {1}00$$

2 $$\% {\text{DL = }}\left[ {{\text{Amount of DCA precipitation NPs }}\left( {{\mathrm{mg}}} \right)/{\text{ Total amount of DCA used }}\left( {{\mathrm{mg}}} \right)} \right] \times {1}00$$

#### Morphological characterization of DCA-PNPs

The DCA-PNPs’ morphological characteristics were assessed by JEOL JEM-2100 High-resolution transmission electron microscopy (TEM) examined. TEM images were analysed via Gatan Digital Micrograph software. Samples were produced by depositing diluted DCA-PNPs in ethanol onto copper grids. A sparse mixture of DCA-PNPs in ethanol was applied to copper surfaces for TEM^70^. The morphology, size, and forms of particle surfaces were examined using a scanning electron microscope (SEM). The nanoparticles were coated via ion sputtering utilising a metallic stub linked to the scanning electron microscope. DCA-PNPs were assessed by a random scan^71^. To enhance imaging quality and minimize aggregation, nanoparticles were coated with a thin gold layer prior to SEM analysis. PVA was used as a stabilizer during nanoparticle synthesis and may form a thin coating layer on the particle surface. Residual PVA, if not completely removed, has been reported to contribute to nanoparticle clustering; thus, washing during ultracentrifugation is essential to minimize this effect.

#### Ultraviolet analysis

The solutions of DCA, DCA-PNPs, and PLGA were formulated at different suitable concentrations (25, 25, 50 µg/mL), respectively. The water bath is linked to the spectrometer to ensure a consistent temperature during the experiment. Spectral measurements entail positioning the sample in a cuvette, configuring the wavelength range (200–300 nm), executing baseline correction, and doing a scan to assess DCA, DCA-PNPs, and PLGA absorption. Data collection includes documenting absorption spectra and identifying wavelengths of maximum absorption (λmax)^72^.

#### Fourier transform infrared spectroscopy analysis (FT-IR)

The (FT-IR) spectra ranging from 4000 to 400 cm^−1^ were obtained using a Perkin Elmer 1720 spectrophotometer on potassium bromide (KBr) plates to investigate the development of intracellular DCA-PNPs^73^.

#### X-ray diffraction patterns (XRD)

The (XRD) spectrum to DCA, DCA-PNPs, and PLGA were examined with a Phillips X PERT-PRO electron microscope for scanning (GNR series, APD2000PRO, Italy) equipped with a monochromatic Cu K (1.5406 Å) X-ray supply. Operating at 48 kV, the device scanned an angle range of 5–35° with a 0.017° step size^74^. The crystal size of DCA, DCA-PNPs, and PLGA were calculated from Scherrer’s equation^75^ at 100% intensity as seen in Eq. (3).

3 $${\mathrm{D}} = (K\lambda /\beta {\text{cos }}\theta )$$

where, D represents particle size, while K is Scherer’s constant (0.94). The wavelength λ of X-rays is 1.54178 Å. β: full width at half maximum (FWHM) for the scattering peak; θ: diffraction angles.

#### Measurements of zeta potential

The zeta electrical potential (ζ) of DCA, DCA-PNPs, and PLGA were assessed utilising a foldable tubular cell and a Zetasizer nano series instrument (Brookhaven, USA). DCA, DCA-PNPs, and PLGA were diluted in distilled water at a concentration of 4% and thereafter introduced into a specialised cuvette. The instrument was calibrated according to the manufacturer’s specifications. An electric field applied to the sample induces the movement of charged nanoparticles. Laser Doppler velocimetry quantifies particle velocity, which is directly associated with zeta potential, derived from the electrophoretic mobility of nanoparticles. Results were presented as an average zeta potential value accompanied by the standard deviation^76^.

### In silico investigations

#### Preparation of ligands

The ligand structures of DCA, Dox, PLGA, and PVA were sourced from the PubChem database in SDF format. Their 3D structures were energy-minimized utilising Avogadro 1.2.0 program with the MMFF94 force field^77^.

#### Protein preparation

The structures of proteins (PDK1-2Q8H, PDK2-4MPN, PDK3-1Y8O, PDK4-7EBB) were retrieved from Protein Data Bank (PDB). Proteins were prepared utilising Molegro Virtual Docker (2008). The Optimized Potentials for Liquid Simulations (OPLS-3) force field was employed to minimize the energy of the protein structures to optimize ligand interactions^77,78^.

#### Molecular docking

Molecular docking analyses were performed using Molegro Virtual Docker (2008) to predict binding processes and affinities of medicines with their corresponding proteins. The docking grid boxes were centred on projected binding sites with a spacing of 15 Å, with the following coordinates: [(PDK1: x = 8.79 Å, y = 27.19 Å, z = 9.66 Å), (PDK2: x = 5.97 Å, y = 15.26 Å, z = 25.99 Å), (PDK3: x = 60.94 Å, y = 0.41 Å, z = 69.47 Å), (PDK4: x = 2.74 Å, y = 0.06 Å, z = 19.04 Å)],^78^.

#### Visualization and analysis

The docked complexes were visualised and analysed using BIOVIA Discovery Studio Visualiser 2020. The binding affinities (ΔG values) and intermolecular interactions, including hydrogen bonds and hydrophobic interactions, were analysed and recorded^77^.

#### ADMET pharmacokinetics features

ADMET pharmacokinetics were performed by online tool ADMETLab2.0 (https://admetmesh.scbdd.com), that provided insight the mechanisms by which these chemicals may operate throughout biological systems^79^.

### In vivo studies

#### Animals and ethical approvements

Seventy CD1 female mice (21 ± 0.5 g) were acquired from animal house husbandry, Alexandria University, Egypt. Mice were acclimatised for one week earlier than to conduct the study under optimal conditions of temperature (23–25 °C), relative humidity (53 ± 4%), and 12-h light/dark cycle. Mice were provided with water and standard pelleted animal feed ad libitum. The study was performed with approvements from the committees of the Faculty of Science, Tanta University under the ethical protocol no. IACUC-SCI-TU-0210, and it adhered to ARRIVE guidelines and the National Institutes of Health requirements guidelines for care and use of laboratory animals (NIH publications no. 8023, revised 1978).

#### Implantation of tumor cells

Ehrlich ascites carcinoma cells were obtained from the National Institute of Cancer at Cairo University, Egypt; and suspended in sterile PBS^80^. Viable cell counts were determined using the trypan blue technique^81^. The cell count was adjusted to 0.5 × 10^6^ cells/mouse for intraperitoneal (*i.p.*) inoculation^82^ in the lower right quadrant of the abdominal cavity using a 25-gauge needle under aseptic conditions. Following inoculation, tumor development was consistently observed as ascitic growth, characterized by progressive abdominal distension, accumulation of ascitic fluid, and increased body weight over.

#### Experimental design

**Fig. 14:**
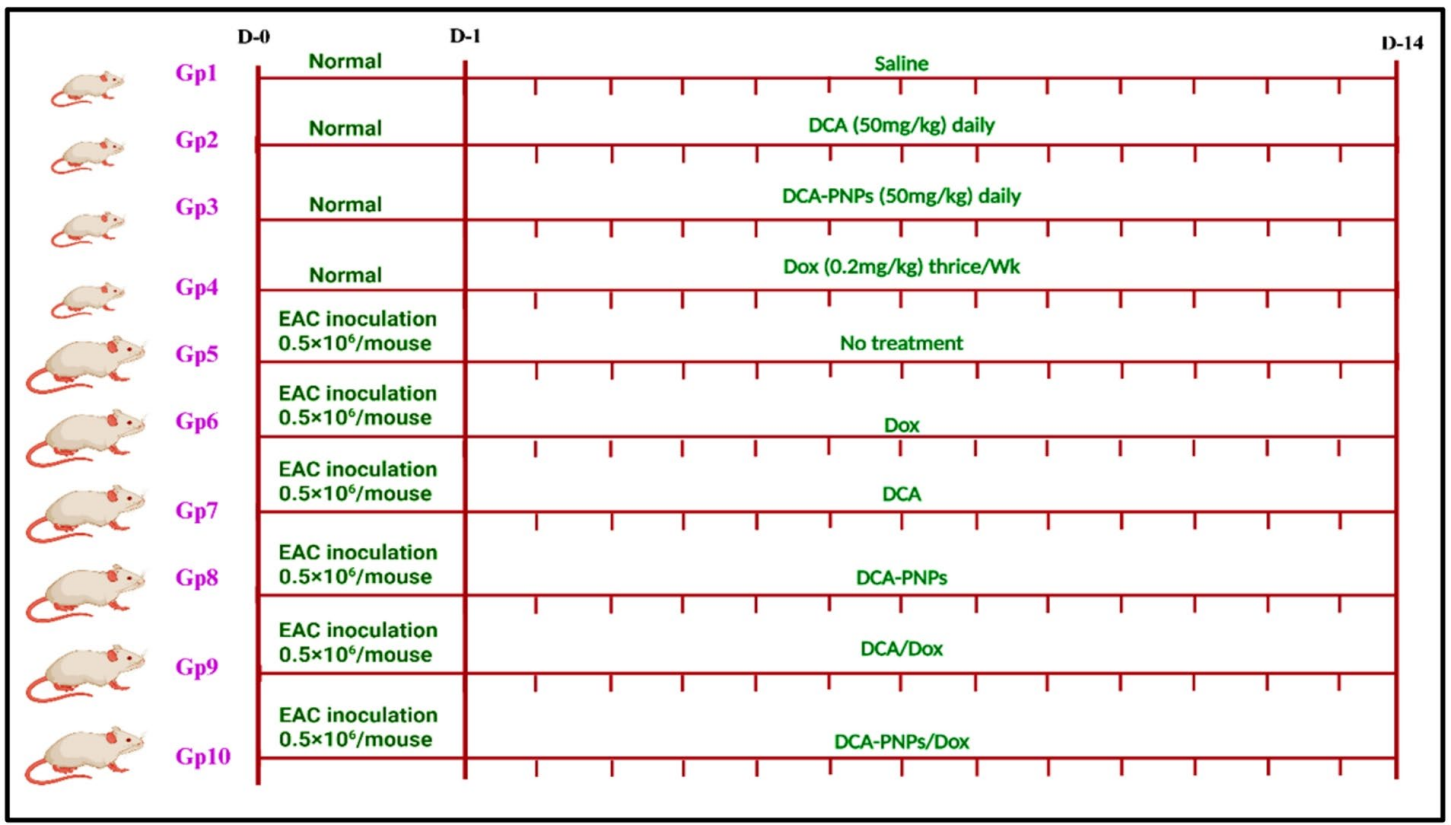
Experimental design illustrates the timeline for normal, EAC bearing mice, and treatment groups for 14 days. Where D: day, Gp: group, and wk: week.

Mice were divided into 10 groups (n = 7), as follows. (Gp1) served as the negative control. Gp2-Gp4 had injected *i.p.* by DCA (50 mg/kg/day), DCA-PNPs (50 mg/kg/day)^83^, and Dox (20 mg/kg) 3 times/week^84^, respectively. Gp5-Gp10 had inoculated *i.p.* with EAC cells (0.5 × 10^6^/mouse). GP5 were untreated group, served as a positive control (EAC-untreated mice or EAC-bearing mice group). Gp6-Gp10 had treated with Dox, DCA, DCA-PNPs, Dox/DCA, and Dox/DCA-PNPs, respectively, the treatments protocol for 14 days as shown in Fig. 14.

#### Sampling

At the end of the experiment, mice were euthanised by sodium barbiturate (300 mg/kg)^85^, sections of liver and kidney tissues were preserved in 10% neutral buffered formalin for histological evaluation. Mitochondria were isolated from tumour cells for the analysis of gene expression.

#### Body weight changes

Body weight (B.wt) of all experimental groups were recorded at day zero (D-0) as initial B.wt (I. B.wt) and at the experiment end as final B.wt (F. B.wt). The body weight change (%) was calculated as Eq. (4) follows^86^.

4 $${\mathrm{B}}.{\text{wt }}\left( \% \right) = \left[ {\left( {{\mathrm{F}}.{\mathrm{B}}.{\text{wt - I}}.{\mathrm{B}}.{\mathrm{wt}}} \right)/\left( {{\mathrm{I}}.{\mathrm{B}}.{\mathrm{wt}}} \right)} \right] \times {1}00$$

#### Mean survival time

Three mice from each group were left under the laboratory condition to calculate the mean survival time (MST), the life span (ILS) and tumor growth inhibition fold change (T/C) were determined according to^87^, as seen in Eqs. (5), (6), and (7).

5 $$\% {\text{MST }} = \left( {\sum {\text{Survival time }}\left( {{\mathrm{days}}} \right){\text{ of each mouse in a group}}} \right)/\left( {\text{Total number of mice}} \right)$$

6 $$\% {\text{ILS }} = \left\{ {\left( {{\text{MST of treated mice}}/{\text{MST of control group}}} \right) - {1}} \right\} \times {1}00$$

7 $$\% {\mathrm{T}}/{\text{C }} = \left( {{\text{MST of treated mice}}/{\text{MST of control group}}} \right) \times {1}00$$

#### Ascitic volume and cell count

In the EAC model, tumour burden was assessed by determining the volume of ascitic fluid and counting the total, viable, and non-viable cells, as this model does not produce a solid tumour mass that can be measured.

Ascitic fluid contained EAC cells was extracted from the peritoneal cavity of each mouse prior to dissection. A graded centrifuge tube was employed to quantify the volume of recovered ascitic fluid. After the suspension of EAC cells in sterile isotonic saline, a Neubauer haemocytometer was employed to enumerate the total, viable, and non-viable EAC cells^88^.

#### Mitochondrial isolation

EAC cells were homogenized in 1 mL of isolation buffer [0.07878 gm Tris–HCL, 0.00186 gm EDTA Disodium, 0.117148 gm sucrose, and 0.4 gm NaCl dissolved in 50 ml Dist.H2O, pH adjusted to 7.4 with 0.05 ml 36% HCl and 0.5 ml 10% NaOH] ice medium; then centrifuged (10 min; 1400 rpm; 4°C) after chilling for 15 min. The supernatant was then centrifuged (15 min; 10,000 rpm; 4°C). Mitochondria were extracted as a precipitate, and the cytosolic component was in the supernatant^89^.

#### Molecular investigations

##### Gene expression using (qRT-PCR)

Pure RNA was extracted from the EAC cells and isolated mitochondria by RNA extraction kit. The quality and quantity of RNA were evaluated by measuring absorbance at 260/280 nm using Nanodrop spectrophotometry (Implen NanoPhotometer®). Messenger RNA (mRNA) was converted into complementary DNA (cDNA) via reverse transcription kits. cDNA was amplified using 2X Maxima SYBR Green/ROX qPCR Master Mix, following the manufacturer’s instructions, to quantify the mRNA expression of target genes (PDK1, 2, 3, and 4), with β-actin as the housekeeping reference gene, as specified in Table 2. Absolute amounts of mRNA were determined utilising the 2^−∆∆Ct^ method^90^.

**Table 2:** Primer design using online software (Home—Nucleotide—NCBI/PrimerQuest PCR & qPCR Primer Design Tool|IDT).

Genes	Forward primer (5`-3`)	Reverse primer (5`-3`)	Accession number
PDK1	AAGAATGAAAAACTGGAATGGGATT	AGAGGCGTGATATGGGCAATCC	NC_000068
PDK2	CTCGCCGTTTACCCATTCCT	TTCAGCAAGTTCTCCCCGTC	NC_000077
PDK3	ATGCTCTCATGTTCACAGAATTAGA	GCAGAGCCATGCGTTTCTTT	NC_000086
PDK4	AGACCGCCTGTTATCCAAGC	ACTGAACACGCTTCACCCAC	NC_000072
β-Actin	GAAGGCTATAGTCACCTCGGG	ATGGTAATAATGCGGCCGGT	NC_000071

##### Apoptotic profile of EAC cells

EAC cells were harvested from EAC-bearing mice that have previously undergone treatment with Dox, DCA, DCA-PNPs, and their combinations. EAC cells underwent two washes with ice-cold PBS, followed by cell density assessment, and were subsequently re-suspended in 1 × annexin-binding buffer to achieve a final density of 1 × 10^6 cells/ml. Subsequently, 100 μl of the cell suspension was transferred into 1.5-ml Eppendorf tubes, followed by the addition of 5 μl of annexin V-fluorescein isothiocyanate (FITC) and 1 μl of propidium iodide (PI) working solution at a concentration of 100 μg/ml. Stained EAC cells were subsequently incubated at room temperature for 15 min, followed by the addition of 400 μl of 1 × annexin-binding solution with gentle mixing; thereafter, the samples were maintained on ice. The cells were subsequently examined using flow cytometry as detailed by^55^.

##### Cell cycle arrest of EAC cells

EAC cells were extracted from EAC-bearing mice that had been previously administered Dox, DCA, DCA-PNPs, and their combinations. EAC cells were produced at a concentration of 2 × 10^6 cells/ml, subjected to two washes with ice-cold PBS, and subsequently fixed in 70% ethanol at 4 °C overnight. The fixed cells were re-suspended in 300–500 μl of a PI/Triton X 100 staining solution (comprising 1000 μl of 0.1% Triton, 40 μl of PI, and 20 μl of RNase) for 30 min at 37 °C in the absence of light. The cells were subsequently centrifuged at 1000 × g, and the cell counts at various phases of the cell cycle were assessed via flow cytometry (BD FACSCanto II, BD Biosciences, USA), with data analysis conducted using BD FACS Diva software^55^.

#### Histopathological assessment

Liver and kidney section tissues were fixed (10% neutral formalin for 24 h). The wax blocks that were implanted were cut into pieces that were 4 m thick. After xylene dewaxing, the slides were stained with haematoxylin and shaken for 30 s. Then, at room temperature (20–25 °C), they were rinsed in water for 1 min and stained with a 1% eosin solution for 30 s while being shaken. For each rat, three paraffin liver slices were prepared to look at changes in the tissues under a microscope (× 400)^91^. Histopathological changes were examined under a light microscope (× 400), and five random high-power fields (HPFs) were analysed per tissue section (n = 5 animals per group). Tumor cell infiltration and tissue lesions were graded semi-quantitatively using ImageJ software on a scale of 0–3: grade 0 = no lesion, grade 1 = mild lesion involving < 25% of the tissue, grade 2 = moderate lesion involving 25–50% of the tissue, and grade 3 = severe lesion involving > 50% of the tissue.

#### Statistical analysis

Results were presented as mean ± SE. All statistical analyses were performed with GraphPad Prism version 6. To assess statistical significance, two-way analyses of variance (ANOVA) were employed. Comparisons were made between groups to demonstrate notable effects of treatment conditions, and *p* ≤ 0.05 was considered statistically significant.

## Conclusion

The NPs formulation of DCA appears to enhance its therapeutic efficacy, while the combination with Dox shows synergistic anti-tumor effects with lethal toxic effects. The treatment modulates PDK expression, induces cell cycle arrest, reduces oxidative stress, and improves overall survival in the EAC mouse model by normalizing biochemical and molecular markers. These findings suggest that the DCA-PNPs-Dox combination therapy may offer a novel approach to targeting cancer cell metabolism and overcoming some limitations of conventional chemotherapy.

## Limitations and future perspectives

The utilization of EAC model might not mimic the complications of mortal BC. Future studies should investigate the impact of Dox/DCA-PNPs combination in different cancer models in vivo and in vitro trials. Furthermore, studies should stand behind the improvement of different nanoparticle formulation for drug delivery, as well as assessing potential of this combination with immunotherapy or radiation therapy.

## Supplementary Information

Supplementary Information.

## Data Availability

The data generated or analysed during the current study are available from the protein data bank (PDK1: 2Q8H, PDK2: 4MPN, PDK3: 1Y8O, PDK4: 7EBB). ADMET prediction analyses were performed by online tool ADMETLab2.0 (https:/admetmesh.scbdd.com), and primer design were performed using Primer design using online software (Home—Nucleotide—NCBI/PrimerQuest [PCR & qPCR Primer Design Tool|IDT]) and checked (No Hair pin formation/No Primer Dimer/No Self-annealing) by these websites (Primer designing tool/Oligonucleotide Properties Calculator/Multiple Primer Analyzer|Thermo Fisher Scientific—EG).

## Abbreviations

ADMET: Absorption, distribution, metabolism, excretion, and toxicity
BC: Breast cancer
B.wt: Body weight
DCA: Dichloroacetate
DCA-PNPs: Dichloroacetate nanoparticles
DL: Drug loading
DNA: Deoxyribonucleic acid
Dox: Doxorubicin
EAC: Ehrlich ascites carcinoma
EE: Encapsulation efficacy
F. B.wt: Final body weight
FTIR: Fourier transform infrared
GP: Group
H&E: Hematoxylin and eosin
HIF-1α: Hypoxia-inducible factor 1α
HPLC: High-performance liquid chromatography
I. B.wt: Initial body weight
ILS: Increase in life span
i.p: Intraperitoneal
MST: Mean survival time
NP: Nanoparticle
PBS: Phosphate buffer saline
PDC: Pyruvate dehydrogenase complex
PDK: Pyruvate dehydrogenase kinase
PI: Propidium iodide
PLGA: Poly D, L-lactic-co-glycolide
PVA: Polyvinyl alcohol
qRT-PCR: Quantitative real-time polymerase chain reaction
RNA: Ribonucleic acid
SE: Standard error
SEM: Scanning electron microscope
T/C: Tumor growth inhibition fold change
TEM: Transmission electron microscopy
UV: Ultraviolet
Wk.: Week
XRD: X-ray diffraction

## References

[1] Liao, L. Inequality in breast cancer: Global statistics from 2022 to 2050. *Breast* **1**(79), 103851. 10.1016/j.breast.2024.103851 (2025). 10.1016/j.breast.2024.103851 PMC11625356 39580931

[2] Elmasry, H. et al. Evaluation of MMP-13 and Micro RNA-138 as prognostic biomarkers for breast cancer in Egyptian women patients. *Pathol. Res. Pract.* **1**(253), 155045. 10.1016/j.prp.2023.155045 (2024). 10.1016/j.prp.2023.155045 38176307

[3] Barba, I., Carrillo-Bosch, L. & Seoane, J. Targeting the Warburg effect in cancer: where do we stand?. *Int. J. Mol. Sci.* **25**(6), 3142. 10.3390/ijms25063142 (2024). 38542116 10.3390/ijms25063142 PMC10970388

[4] Dong, X. M. et al. Exploring metabolic reprogramming in esophageal cancer: the role of key enzymes in glucose, amino acid, and nucleotide pathways and targeted therapies. *Cancer Gene Ther.* **32**(2), 165–183. 10.1038/s41417-024-00858-5 (2025). 39794467 10.1038/s41417-024-00858-5

[5] Koltai, T. & Fliegel, L. Dichloroacetate for cancer treatment: some facts and many doubts. *Pharmaceuticals.* **17**(6), 744. 10.3390/ph17060744 (2024). 38931411 10.3390/ph17060744 PMC11206832

[6] Aboslema, R. F. Role of glycolysis in breast cancer. *Rec. Pharm. Biomed. Sci.* **8**(3), 1. 10.21608/rpbs.2024.256835.1258 (2024).

[7] Kobayashi, H., Matsubara, S., Yoshimoto, C., Shigetomi, H. & Imanaka, S. The role of mitochondrial dynamics in the pathophysiology of endometriosis. *J. Obstetrics Gynaecol. Res.* **49**(12), 2783–2791. 10.1111/jog.15791 (2023). 10.1111/jog.15791 37681703

[8] Alberghina, L. The Warburg effect explained: Integration of enhanced glycolysis with heterogeneous mitochondria to promote cancer cell proliferation. *Int. J. Mol. Sci.* **24**(21), 15787. 10.3390/ijms242115787 (2023). 37958775 10.3390/ijms242115787 PMC10648413

[9] Mirzaei, S., Ranjbar, B., Tackallou, S. H. & Aref, A. R. Hypoxia inducible factor-1α (HIF-1α) in breast cancer: The crosstalk with oncogenic and onco-suppressor factors in regulation of cancer hallmarks. *Pathol. Res. Pract.* **1**(248), 154676. 10.1016/j.prp.2023.154676 (2023). 10.1016/j.prp.2023.154676 37454494

[10] Saleh, N., Allam, T., Abdelfattah, A. & El-Borai, N. Review on Ehrlich Ascites Carcinoma in mice and cancer treatment with special reference to the potential protective and therapeutic effects of hesperidin versus cisplatin. *J. Current Veterinary Res.* **4**(1), 47–57. 10.21608/jcvr.2022.240866 (2022).

[11] Eltahir, Z., Ibrahim, M., Mohieldeen, M. Y., Bayoumi, A. & Ahmed, S. M. Thymoquinone nanoparticles (TQ-NPs) in kidney toxicity induced by ehrlich ascites carcinoma (EAC): An in vivo study. *Can. J. Kidney Health Dis.* **11**, 20543581241258812. 10.1177/20543581241258812 (2024). 38863503 10.1177/20543581241258812 PMC11165950

[12] Gomaa, S., Nassef, M., Tabl, G., Zaki, S. & Abdel-Ghany, A. Doxorubicin and folic acid-loaded zinc oxide nanoparticles-based combined anti-tumor and anti-inflammatory approach for enhanced anti-cancer therapy. *BMC Cancer* **24**(1), 34. 10.1186/s12885-023-11714-4 (2024). 38178054 10.1186/s12885-023-11714-4 PMC10768430

[13] Khatun, N. et al. Green synthesis of silver/silver chloride nanoparticles derived from Elaeocarpus floribundus leaf extract and study of its anticancer potential against EAC and MCF-7 cells with antioxidant and antibacterial properties. *Res. Chem.* **1**(7), 101287. 10.1016/j.rechem.2023.101287 (2024).

[14] Mansour MS, Mahmoud AA, Sayah MA, Mohamed ZN, Hussein MA, ALsherif DA. RES-CMCNPs enhance antioxidant, proinflammatory, and sensitivity of tumor solids to γ-irradiation in EAC-bearing mice. Pharmaceutical Nanotechnology. 2025 Feb;13(1):254–69. 10.2174/0122117385290497240324190453 10.2174/0122117385290497240324190453 38676484

[15] Sheibani, M. et al. Doxorubicin-induced cardiotoxicity: An overview on pre-clinical therapeutic approaches. *Cardiovasc. Toxicol.* **22**(4), 292–310. 10.1007/s12012-022-09721-1 (2022). 35061218 10.1007/s12012-022-09721-1

[16] Rahmani, E. et al. Preparation of a pH-responsive chitosan-montmorillonite-nitrogen-doped carbon quantum dots nanocarrier for attenuating doxorubicin limitations in cancer therapy. *Eng. Life Sci.* **22**(10), 634–649. 10.1002/elsc.202200016 (2022). 36247828 10.1002/elsc.202200016 PMC9550734

[17] Setia, A. et al. Nanomedicine and nanotheranostics: special focus on imaging of anticancer drugs induced cardiac toxicity. *Nanotheranostics.* **8**(4), 473. 10.7150/ntno.96846 (2024). 38961885 10.7150/ntno.96846 PMC11217786

[18] Tufail, M., Jiang, C. H. & Li, N. Altered metabolism in cancer: Insights into energy pathways and therapeutic targets. *Mol. Cancer* **23**(1), 203. 10.1186/s12943-024-02119-3 (2024). 39294640 10.1186/s12943-024-02119-3 PMC11409553

[19] Bianchi C, Martinelli RP, Rozados VR, Scharovsky OG. Use of sodium dichloroacetate for cancer treatment: A scoping review. http://hdl.handle.net/11336/238350 38683516

[20] Khan ZF, Rathi A, Khan A, Anjum F, Chaudhury A, Taiyab A, Shamsi A, Hassan MI. Discovering Therapeutic Candidates for Lung Cancer via PDK3 Inhibition–A drug repurposing approach. 10.21203/rs.3.rs-4795408/v1

[21] Bufalini, C. et al. Encapsulation of Arthrospira platensis polyphenolic extract using supercritical emulsion-based process. *J. Supercrit. Fluids.* **1**(212), 106335. 10.1016/j.supflu.2024.106335 (2024).

[22] Pandey, G. et al. ‘Nano-in-nano’–Breaching the barriers of the tumor microenvironment using nanoparticle-incorporated nanofibers. *J. Drug Deliv. Sci. Technol.* **1**(91), 105249. 10.1016/j.jddst.2023.105249 (2024).

[23] Zhuo, Y., Zhao, Y. G. & Zhang, Y. Enhancing drug solubility, bioavailability, and targeted therapeutic applications through magnetic nanoparticles. *Molecules* **29**(20), 4854. 10.3390/molecules29204854 (2024). 39459222 10.3390/molecules29204854 PMC11510236

[24] Yadav, P. K. et al. Ratiometric codelivery of Paclitaxel and Baicalein loaded nanoemulsion for enhancement of breast cancer treatment. *Int. J. Pharm.* **25**(643), 123209. 10.1016/j.ijpharm.2023.123209 (2023). 10.1016/j.ijpharm.2023.123209 37422142

[25] Trapella, C. et al. Design, synthesis, and biological characterization of novel mitochondria targeted dichloroacetate-loaded compounds with antileukemic activity. *J. Med. Chem.* **59**(1), 147–156. 10.1021/acs.jmedchem.5b01165 (2016). 26653539 10.1021/acs.jmedchem.5b01165

[26] Todaro, B., Moscardini, A. & Luin, S. Pioglitazone-loaded PLGA nanoparticles: Towards the most reliable synthesis method. *Int. J. Mol. Sci.* **23**(5), 2522. 10.3390/ijms23052522 (2022). 35269665 10.3390/ijms23052522 PMC8910508

[27] Mostafa, M. M. et al. Chitosan surface-modified PLGA nanoparticles loaded with cranberry powder extract as a potential oral delivery platform for targeting colon cancer cells. *Pharmaceutics.* **15**(2), 606. 10.3390/pharmaceutics15020606 (2023). 36839928 10.3390/pharmaceutics15020606 PMC9964659

[28] Shiraz, M., Imtiaz, H., Azam, A. & Hayat, S. Phytogenic nanoparticles: Synthesis, characterization, and their roles in physiology and biochemistry of plants. *Biometals* **37**(1), 23–70. 10.1007/s10534-023-00542-5 (2024). 37914858 10.1007/s10534-023-00542-5

[29] Gharbavi, M., Johari, B., Tabar, R. M. & Sharafi, A. Selenium-doped albumin nanoparticles enhance tamoxifen-induced anticancer effects in 4T–1 mouse breast cancer cells. *Appl. Organomet. Chem.* **38**(2), e7327. 10.1002/aoc.7327 (2024).

[30] Carreón González, J. L., García Casillas, P. E. & Chapa, G. C. Gold nanoparticles as drug carriers: The role of silica and peg as surface coatings in optimizing drug loading. *Micromachines.* **14**(2), 451. 10.3390/mi14020451 (2023). 36838151 10.3390/mi14020451 PMC9965813

[31] Patel, M., Mishra, S., Verma, R. & Shikha, D. Synthesis of ZnO and CuO nanoparticles via Sol gel method and its characterization by using various technique. *Discov. Mater.* **2**(1), 1. 10.1007/s43939-022-00022-6 (2022).

[32] Tahamtan, S. et al. Biocompatible chitosan/starch/graphene quantum dots/titanium dioxide nanocomposite: A stimuli-responsive, porous nanocarrier for prolonged quercetin delivery in lung cancer treatment. *BioNanoScience.* **14**(3), 2491–2508. 10.1007/s12668-024-01461-6 (2024).

[33] Al Shidi, W., Ganat, T., Lashari, N., Taura, U. & Kazemi, A. Innovations in polymeric nanofluid technologies for enhanced oil recovery: A comprehensive review. *Arab. J. Sci. Eng.* **10**, 1–27. 10.1007/s13369-025-10166-1 (2025).

[34] Qi, Y. et al. The associated killing of hepatoma cells using multilayer drug-loaded mats combined with fast neutron therapy. *Nano Res.* **14**(3), 778–787. 10.1007/s12274-020-3113-1 (2021).

[35] Gautam, S., Pathak, S. & Dubey, S. H. The role of molecular docking in modern drug discovery and development: A comprehensive review. *J. Drug Discov. Health Sci.* **1**(03), 129–137. 10.21590/jddhs.01.03.02 (2024).

[36] She, W. et al. Reprogramming energy metabolism with synthesized PDK inhibitors based on dichloroacetate derivatives and targeted delivery systems for enhanced cancer therapy. *J. Med. Chem.* **66**(21), 14683–14699. 10.1021/acs.jmedchem.3c01197 (2023). 37688544 10.1021/acs.jmedchem.3c01197

[37] Chen, T. et al. Inhibition of pyruvate dehydrogenase kinase 4 protects cardiomyocytes from lipopolysaccharide-induced mitochondrial damage by reducing lactate accumulation. *Inflammation* **47**(4), 1356–1370. 10.1007/s10753-024-01981-z (2024). 38401019 10.1007/s10753-024-01981-z

[38] Sucharitha P, Reddy KR, Satyanarayana SV, Garg T. Absorption, distribution, metabolism, excretion, and toxicity assessment of drugs using computational tools. InComputational approaches for novel therapeutic and diagnostic designing to mitigate SARS-CoV-2 infection 2022 Jan 1 (pp. 335–355). Academic Press. 10.1016/B978-0-323-91172-6.00012-1

[39] Sonaxi, S. A., Afshari, M. & Tomar, R. Unveiling the anticancer potential of Formylbenzyl-N N-Dimethylmethanaminium-based ionic liquids via cheminformatics approaches. *J. Chem.* **2025**(1), 6967930. 10.1155/joch/6967930 (2025).

[40] Prasad, S. R. et al. Doxorubicin-Polysorbate 80 conjugates: targeting effective and sustained delivery to the brain. *RSC Pharmaceutics.* **1**(3), 412–429. 10.1039/D4PM00053F (2024).

[41] Soltani, A. et al. Improvement of anti-inflammatory and anticancer activities of poly (lactic-co-glycolic acid)-sulfasalazine microparticle via density functional theory, molecular docking and ADMET analysis. *Arab. J. Chem.* **15**(1), 103464. 10.1016/j.arabjc.2021.103464 (2022).

[42] Tuba, R., Al-Hashimi, M., Bazzi, H. S. & Grubbs, R. H. One-pot synthesis of poly (vinyl alcohol)(PVA) copolymers via ruthenium catalyzed equilibrium ring-opening metathesis polymerization of hydroxyl functionalized cyclopentene. *Macromolecules* **47**(23), 8190–8195. 10.1021/ma501976v (2014).

[43] Patil, P. P. et al. Computational and experimental pharmacology to decode the efficacy of Theobroma cacao L. against doxorubicin-induced organ toxicity in EAC-mediated solid tumor-induced mice. *Front. Pharmacol.* **31**(14), 1174867. 10.3389/fphar.2023.1174867 (2023). 10.3389/fphar.2023.1174867 PMC10264642 37324470

[44] Perše, M. Cisplatin mouse models: treatment, toxicity and translatability. *Biomedicines.* **9**(10), 1406. 10.3390/biomedicines9101406 (2021). 34680523 10.3390/biomedicines9101406 PMC8533586

[45] Hefny, S. M. et al. Discovery and mechanistic studies of dual-target hits for carbonic anhydrase IX and VEGFR-2 as potential agents for solid tumors: X-ray, in vitro, in vivo, and in silico investigations of coumarin-based thiazoles. *J. Med. Chem.* **67**(9), 7406–7430. 10.1021/acs.jmedchem.4c00239 (2024). 38642371 10.1021/acs.jmedchem.4c00239

[46] Shete, M. B., Deshpande, A. S. & Shende, P. Silybin-based herbal nanocarriers: an advancement in anticancer therapy. *Mater. Technol.* **37**(13), 2832–2852. 10.1080/10667857.2022.2081286 (2022).

[47] Duarte, D. & Vale, N. Evaluation of synergism in drug combinations and reference models for future orientations in oncology. *Current Res. Pharmacol. Drug Discov.* **1**(3), 100110. 10.1016/j.crphar.2022.100110 (2022). 10.1016/j.crphar.2022.100110 PMC9127325 35620200

[48] Weiner, F. et al. Evaluation of combination protocols of the chemotherapeutic agent FX-9 with azacitidine, dichloroacetic acid, doxorubicin or carboplatin on prostate carcinoma cell lines. *PLoS ONE* **16**(8), e0256468. 10.1371/journal.pone.0256468 (2021). 34432846 10.1371/journal.pone.0256468 PMC8386839

[49] Tao, S., Tao, K. & Cai, X. Pan-cancer analysis reveals PDK family as potential indicators related to prognosis and immune infiltration. *Sci. Rep.* **14**(1), 5665. 10.1038/s41598-024-55455-1 (2024). 38453992 10.1038/s41598-024-55455-1 PMC10920909

[50] Nuyttens, L., Vandewalle, J. & Libert, C. Sepsis-induced changes in pyruvate metabolism: insights and potential therapeutic approaches. *EMBO Mol. Med.* **16**(11), 2678–2698. 10.1038/s44321-024-00155-6 (2024). 39468303 10.1038/s44321-024-00155-6 PMC11554794

[51] Wang, N. et al. The landscape of PDK1 in breast cancer. *Cancers* **14**(3), 811. 10.3390/cancers14030811 (2022). 35159078 10.3390/cancers14030811 PMC8834120

[52] Dwyer, A. R. et al. Glucocorticoid receptors drive breast cancer cell migration and metabolic reprogramming via PDK4. *Endocrinology* **164**(7), bqad083. 10.1210/endocr/bqad083 (2023). 37224504 10.1210/endocr/bqad083 PMC10251300

[53] Zhou, Y., Guo, Y. & Tam, K. Y. Targeting glucose metabolism to develop anticancer treatments and therapeutic patents. *Expert Opin. Ther. Pat.* **32**(4), 441–453. 10.1080/13543776.2022.2027912 (2022). 35001793 10.1080/13543776.2022.2027912

[54] Gong, F., Jin, J., Li, H. & Mao, H. RETRACTED ARTICLE: Alpha-lipoic acid protects against doxorubicin-induced cardiotoxicity by regulating pyruvate dehydrogenase kinase 4. *Cardiovasc. Toxicol.* **22**(10), 879–891. 10.1007/s12012-022-09766-2 (2022). 35930219 10.1007/s12012-022-09766-2

[55] Salem, M. M., Youssef, N. S., El Keiy, M. & Khamis, A. A. The mitigated effect of the combination of metformin and stearic acid to ameliorate bleomycin-induced pulmonary fibrosis in rats via inhibiting Gal-3/Smad3/α-SMA and TNF-α/NF-κβ signaling pathways. *J. Biochem. Mol. Toxicol.* **39**(9), e70494. 10.1002/jbt.70494 (2025). 40952799 10.1002/jbt.70494

[56] Lieschke, E. et al. Flow cytometric single cell-based assay to simultaneously detect cell death, cell cycling, DNA content and cell senescence. *Cell Death Differ.* **29**(5), 1004–1012. 10.1038/s41418-022-00964-7 (2022). 35264779 10.1038/s41418-022-00964-7 PMC9091206

[57] Bogdanov, A. et al. Tumor alkalization therapy: misconception or good therapeutics perspective?–the case of malignant ascites. *Front. Oncol.* **8**(14), 1342802. 10.3389/fonc.2024.1342802 (2024). 10.3389/fonc.2024.1342802 PMC10881708 38390269

[58] Ligasová, A., Frydrych, I. & Koberna, K. Basic methods of cell cycle analysis. *Int. J. Mol. Sci.* **24**(4), 3674. 10.3390/ijms24043674 (2023). 36835083 10.3390/ijms24043674 PMC9963451

[59] Hasannia M, Lamei K, Abnous K, Taghdisi SM, Nekooei S, Nekooei N, Ramezani M, Alibolandi M. Targeted poly (L-glutamic acid)-based hybrid peptosomes co-loaded with doxorubicin and USPIONs as a theranostic platform for metastatic breast cancer. Nanomed. Nanotechnol. Biol. Med. 2023;48:102645. 10.1016/j.nano.2022.102645 10.1016/j.nano.2022.102645 36549556

[60] Famta, P. et al. Exploration of multi-layered nanofiber adjuvant implants of doxorubicin and resveratrol to prevent post-surgery tumor recurrence and invasion. *J. Drug Deliv. Sci. Technol.* **1**(99), 105977. 10.1016/j.jddst.2024.105977 (2024).

[61] Safwat, G., Soliman, E. S. & Mohamed, H. R. Induction of ROS mediated genomic instability, apoptosis and G0/G1 cell cycle arrest by erbium oxide nanoparticles in human hepatic Hep-G2 cancer cells. *Sci. Rep.* **12**(1), 16333. 10.1038/s41598-022-20830-3 (2022). 36175500 10.1038/s41598-022-20830-3 PMC9522848

[62] Alwaili, M. A. et al. Avenanthramide-C ameliorate doxorubicin-induced hepatotoxicity via modulating Akt/GSK-3β and Wnt-4/β-Catenin pathways in male rats. *Front. Mol. Biosci.* **2**(11), 1507786. 10.3389/fmolb.2024.1507786 (2024). 10.3389/fmolb.2024.1507786 PMC11646862 39687571

[63] Elwan, M. M., Elserafy, M. A., Elborady, O. M., Massoud, A. & Aboushafei, A. The potential therapeutic effect of loaded ginger nanoparticles against Ehrlich ascites carcinoma (EAC) bearing mice-induced renal toxicity. *Delta J. Sci.* **48**(2), 142–160. 10.21608/djs.2024.310411.1180 (2024).

[64] Gowda, N. G. et al. Ehrlich Ascites carcinoma mice model for studying liver inflammation and fibrosis. *Adv. Cancer Biol. Metastasis.* **1**(4), 100029. 10.1016/j.adcanc.2022.100029 (2022).

[65] Arrigoni, R., Jirillo, E. & Caiati, C. Pathophysiology of doxorubicin-mediated cardiotoxicity. *Toxics.* **13**(4), 277. 10.3390/toxics13040277 (2025). 40278593 10.3390/toxics13040277 PMC12031459

[66] Song, X. et al. Thiolated chitosan nanoparticles for stable delivery and smart release of As2O3 for liver cancer through dual actions. *Carbohyd. Polym.* **1**(303), 120462. 10.1016/j.carbpol.2022.120462 (2023). 10.1016/j.carbpol.2022.120462 36657859

[67] Xing, H. et al. Redox and pH dual-responsive polypeptide micelles for doxorubicin delivery with enhanced anticancer efficacy. *ACS Appl. Polymer Mater.* **5**(5), 3717–3727. 10.1021/acsapm.3c00371 (2023).

[68] Schoenmann N, Tannenbaum N, Hodgeman RM, Raju RP. Regulating mitochondrial metabolism by targeting pyruvate dehydrogenase with dichloroacetate, a metabolic messenger. Biochimica et Biophysica Acta (BBA)-Molecular Basis of Disease. 2023;1869(7):166769. 10.1016/j.bbadis.2023.166769 10.1016/j.bbadis.2023.166769 PMC10776176 37263447

[69] Alkholief, M., Kalam, M. A., Anwer, M. K. & Alshamsan, A. Effect of solvents, stabilizers and the concentration of stabilizers on the physical properties of poly (D, L-lactide-co-glycolide) nanoparticles: Encapsulation, in vitro release of indomethacin and cytotoxicity against HepG2-cell. *Pharmaceutics.* **14**(4), 870. 10.3390/pharmaceutics14040870 (2022). 35456705 10.3390/pharmaceutics14040870 PMC9028368

[70] El-Demerdash, F. M., Al Mhanna, A. B., El-Sayed, R. A., Mohamed, T. M. & Salem, M. M. Hepatoprotective impact of Nigella sativa silver nanocomposite against genotoxicity, oxidative stress, and inflammation induced by thioacetamide. *Tissue Cell.* **87**, 102332. 10.1016/j.tice.2024.102332 (2024). 38367325 10.1016/j.tice.2024.102332

[71] El-Nahass, M. N. et al. Functionalized gold nanorods turn-on chemosensor for selective detection of Cd2+ ions, bio-imaging, and antineoplastic evaluations. *J. Iran. Chem. Soc.* **21**(3), 699–718. 10.1007/s13738-023-02952-1 (2024).

[72] Menon, A. H. et al. Sustained release of chrysin from chitosan-based scaffolds promotes mesenchymal stem cell proliferation and osteoblast differentiation. *Carbohyd. Polym.* **1**(195), 356–367. 10.1016/j.carbpol.2018.04.115 (2018). 10.1016/j.carbpol.2018.04.115 29804987

[73] Yeom, D., Govindan, M. & Kim, D. Electrocatalytic reduction of gaseous dichloroethane using carbon organic frame immobilized graphene. *J. Electroanal. Chem.* **1**(970), 118539. 10.1016/j.jelechem.2024.118539 (2024).

[74] Boparai, H. K., El-Sharnouby, O. & O’Carroll, D. M. Catalytic dechlorination of 1, 2-DCA in nano Cu0-borohydride system: Effects of Cu0/Cun+ ratio, surface poisoning, and regeneration of Cu0 sites. *Sci. Rep.* **13**(1), 11883. 10.1038/s41598-023-38678-6 (2023). 37482593 10.1038/s41598-023-38678-6 PMC10363550

[75] Vinila VS, Isac J. Synthesis and structural studies of superconducting perovskite GdBa2Ca3Cu4O10. 5+ δ nanosystems. InDesign, fabrication, and characterization of multifunctional nanomaterials 2022 Jan 1 (pp. 319–341). Elsevier. 10.1016/B978-0-12-820558-7.00022-4

[76] Salim, E. I., Mosbah, A. M., Elhussiny, F. A., Hanafy, N. A. & Abdou, Y. Preparation and characterization of cetuximab-loaded egg serum albumin nanoparticles and their uses as a drug delivery system against Caco-2 colon cancer cells. *Cancer Nanotechnol.* **14**(1), 4. 10.1186/s12645-022-00153-8 (2023).

[77] Hanwell, M. D. et al. Avogadro: an advanced semantic chemical editor, visualization, and analysis platform. *J. Cheminform.* **4**(1), 17. 10.1186/1758-2946-4-17 (2012). 22889332 10.1186/1758-2946-4-17 PMC3542060

[78] Harder, E. et al. OPLS3: a force field providing broad coverage of drug-like small molecules and proteins. *J. Chem. Theory Comput.* **12**(1), 281–296. 10.1021/acs.jctc.5b00864 (2016). 26584231 10.1021/acs.jctc.5b00864

[79] Noser, A. A., El-Barbary, A. A., Salem, M. M., El Salam, H. A. A. & Shahien, M. Synthesis and molecular docking simulations of novel azepines based on quinazolinone moiety as prospective antimicrobial and antitumor hedgehog signaling inhibitors. *Sci. Rep.* **14**(1), 3530. 10.1038/s41598-024-53517-y (2024). 38347004 10.1038/s41598-024-53517-y PMC10861550

[80] Aldayel, T. S. et al. Evaluation of antioxidant, anti-inflammatory, anticancer activities and molecular docking of Moringa oleifera seed oil extract against experimental model of Ehrlich ascites carcinoma in Swiss female albino mice. *BMC Compl. Med. Therap.* **23**(1), 457. 10.1186/s12906-023-04279-z (2023). 10.1186/s12906-023-04279-z PMC10720142 38098043

[81] Bhat MA, Khan FA, Kataria HC. In-vivo Anticancer Activity of Root and Leaf extract of Jurinea dolomiaea Boiss (Asteraceae) against Ehrlichs Ascites Carcinoma (EAC) Cell Line. Journal of Advanced Chemical Sciences. 2022 Jul 23:770–3. 10.30799/jacs.244.22080201

[82] Tayel, F., Mahfouz, M. E., Salama, A. F. & Mansour, M. A. Ethoxyquin inhibits the progression of Murine Ehrlich Ascites Carcinoma through the inhibition of autophagy and LDH. *Biomedicines.* **9**(11), 1526. 10.3390/biomedicines9111526 (2021). 34829755 10.3390/biomedicines9111526 PMC8615101

[83] Gonzalez-Leon, A., Schultz, I. R., Xu, G. & Bull, R. J. Pharmacokinetics and metabolism of dichloroacetate in the F344 rat after prior administration in drinking water. *Toxicol. Appl. Pharmacol.* **146**(2), 189–195. 10.1006/taap.1997.8232 (1997). 9344886 10.1006/taap.1997.8232

[84] Ren, S. et al. Comparison of pharmacokinetics, tissue distribution and pharmacodynamics of liposomal and free doxorubicin in tumour-bearing mice following intratumoral injection. *J. Pharm. Pharmacol.* **66**(9), 1231–1239. 10.1111/jphp.12257 (2014). 24716458 10.1111/jphp.12257

[85] Kramer KJ, Ganzberg SI. Pharmacology of Outpatient Anesthesia Medications. InPeterson’s Principles of Oral and Maxillofacial Surgery 2022 Aug 9 (pp. 53–80). Cham: Springer International Publishing. 10.1007/978-3-030-91920-7_3

[86] Wankhede, S. P., Alshehri, A. H. & Du, X. Encapsulating and inkjet-printing flexible conductive patterns on a fluoroelastomer for harsh hydrocarbon fluid environments. *J. Mater. Chem. C.* **11**(12), 3964–3980. 10.1039/D2TC04218E (2023).

[87] Elsherbiny, N. M., Younis, N. N., Shaheen, M. A. & Elseweidy, M. M. The synergistic effect between vanillin and doxorubicin in Ehrlich ascites carcinoma solid tumor and MCF-7 human breast cancer cell line. *Pathol Res Pract.* **212**(9), 767–777. 10.1016/j.prp.2016.06.004 (2016). 27493101 10.1016/j.prp.2016.06.004

[88] Attia, A. A. et al. Amygdalin potentiates the anti-cancer effect of Sorafenib on Ehrlich ascites carcinoma and ameliorates the associated liver damage. *Sci. Rep.* **12**(1), 6494. 10.1038/s41598-022-10517-0 (2022). 35444229 10.1038/s41598-022-10517-0 PMC9021277

[89] Ding, J. et al. Soy isoflavone attenuates brain mitochondrial oxidative stress induced by beta-amyloid peptides 1–42 injection in lateral cerebral ventricle. *J. Neurosci. Res.* **91**(4), 562–567. 10.1002/jnr.23163 (2013). 23239252 10.1002/jnr.23163

[90] Livak, K. J. & Schmittgen, T. D. Analysis of relative gene expression data using real-time quantitative PCR and the 2−^ΔΔ^^CT^ method. *Methods* **25**(4), 402–408. 10.1006/meth.2001.1262 (2001). 11846609 10.1006/meth.2001.1262

[91] Basyoni, A. E., Atta, A., Salem, M. M. & Mohamed, T. M. Harnessing exosomes for targeted drug delivery systems to combat brain cancer. *Cancer Cell Int.* **25**(1), 150. 10.1186/s12935-025-03731-z (2025). 40234973 10.1186/s12935-025-03731-z PMC12001718

