# Supplementary material for: Synergistic therapeutic impact of dichloroacetate nanoparticles and doxorubicin in modulating pyruvate dehydrogenase kinase in breast carcinoma model
Source: Sci Rep. 2026 Jan 23;16:3267. doi: 10.1038/s41598-025-34562-7 (PMC12835023; doi:10.1038/s41598-025-34562-7)
Supplement: Supplementary file 1 — Supplementary Information. [file 41598_2025_34562_MOESM1_ESM.pdf]

Supplementary Information

Synergistic therapeutic impact of dichloroacetate nanoparticles and doxorubicin in modulating pyruvate dehydrogenase kinase in breast carcinoma model.

Maha M. Salem[1*], Amira T. Khattab[1,2], Doha M. Beltagy[2], Mai M. El-Keiy[1]

[1]Biochemistry Division, Chemistry Department, Faculty of Science, Tanta University, Tanta 31257, Egypt

[2]Biochemistry Department, Faculty of Science, Damanhour University, Damnhour 22514, Egypt

***Correspondence & proof**

Dr. Maha M. Salem,

Biochemistry Division, Chemistry Department, Faculty of Science, Tanta University, Tanta, Egypt.

E-mail: maha_salem@science.tanta.edu.eg

ORCID ID: 0000-0002-9108-6932

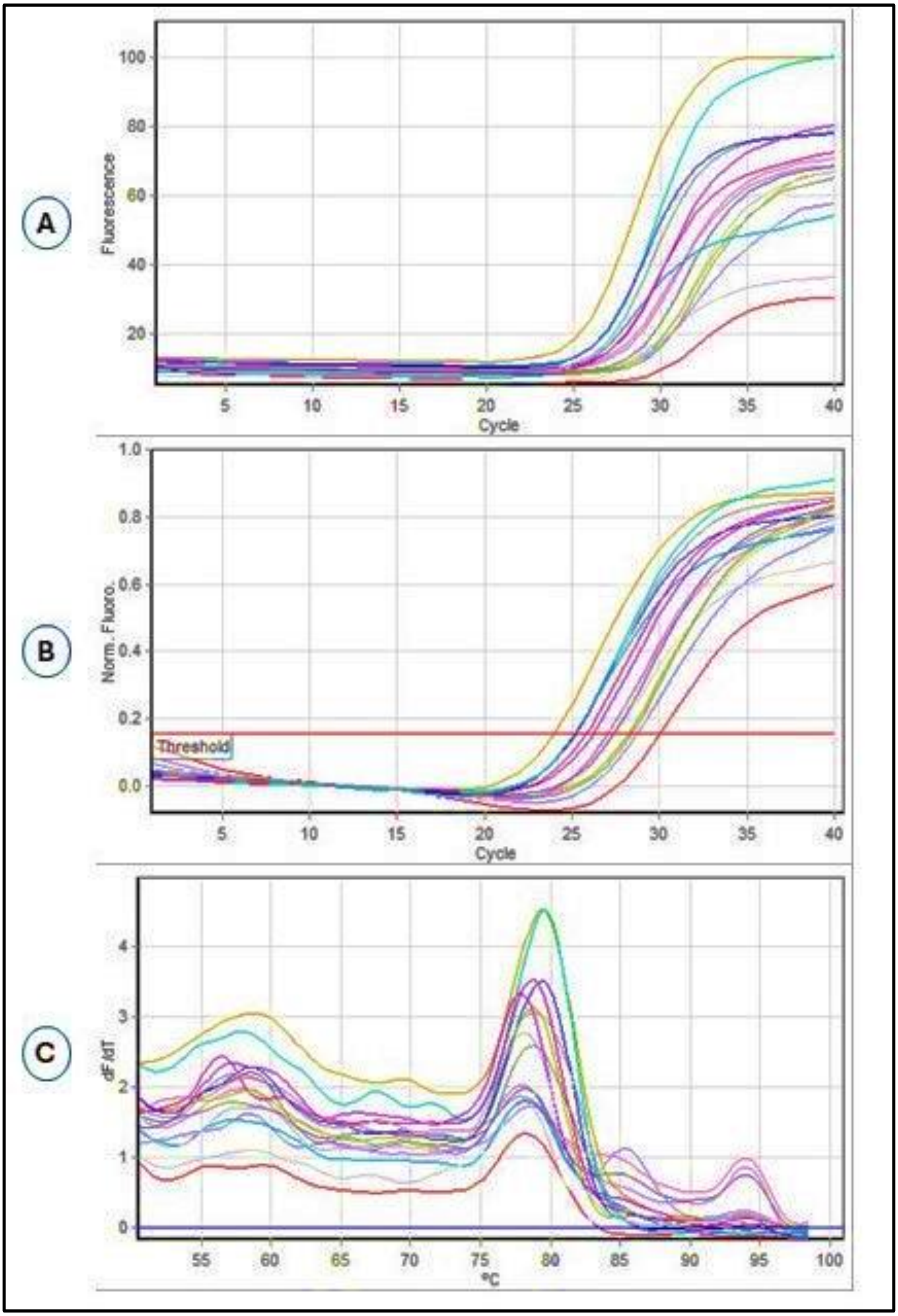

Figure S1: (A) The linear (upper), **(B)** log (lower) amplification curves representing the Ct values and **(C)** melting curves of β-actin gene in all experimental groups

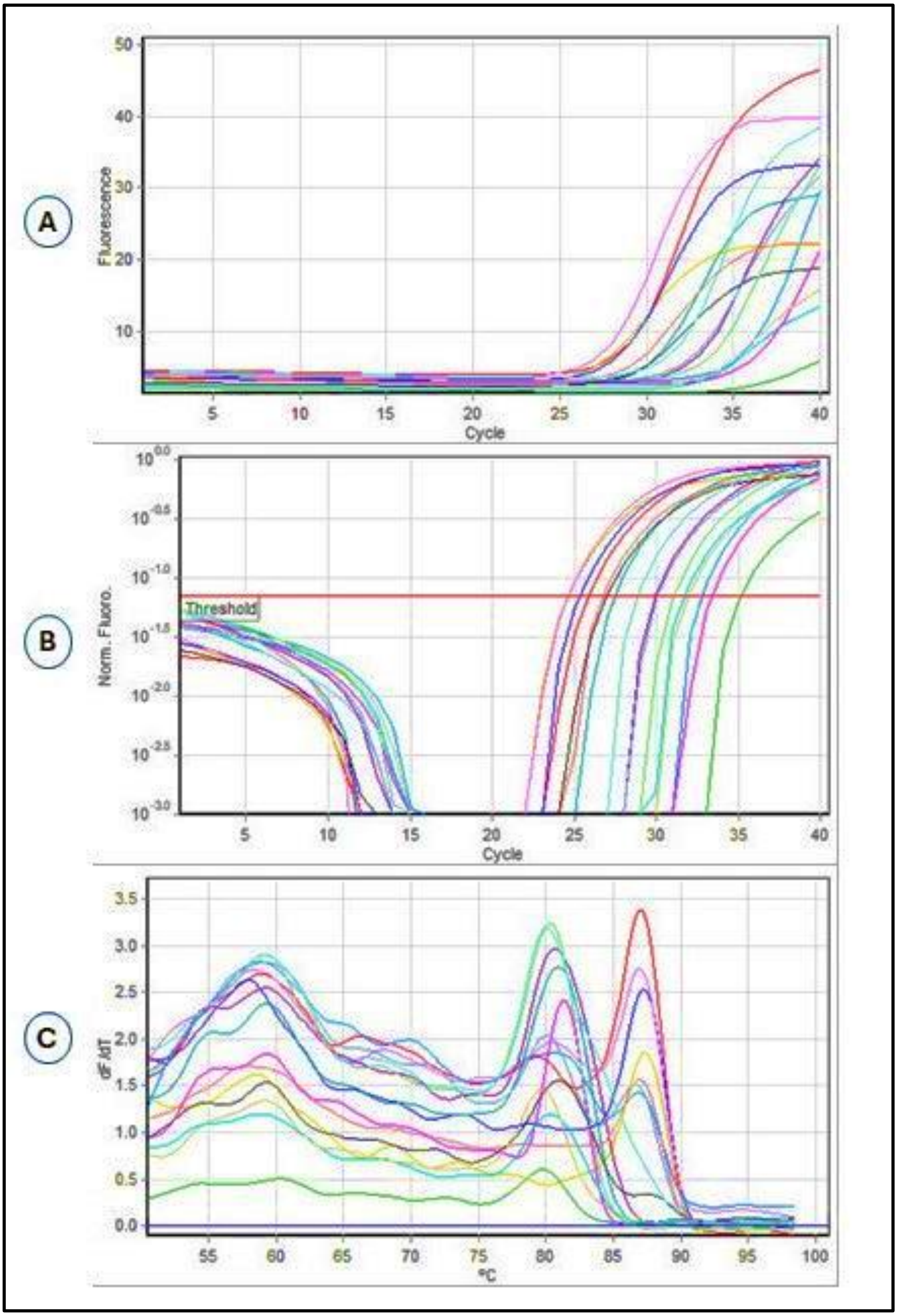

Figure S2: (A) The linear (upper), **(B)** log (lower) amplification curves representing the Ct values and **(C)** melting curves of PDK-1 gene in all experimental groups

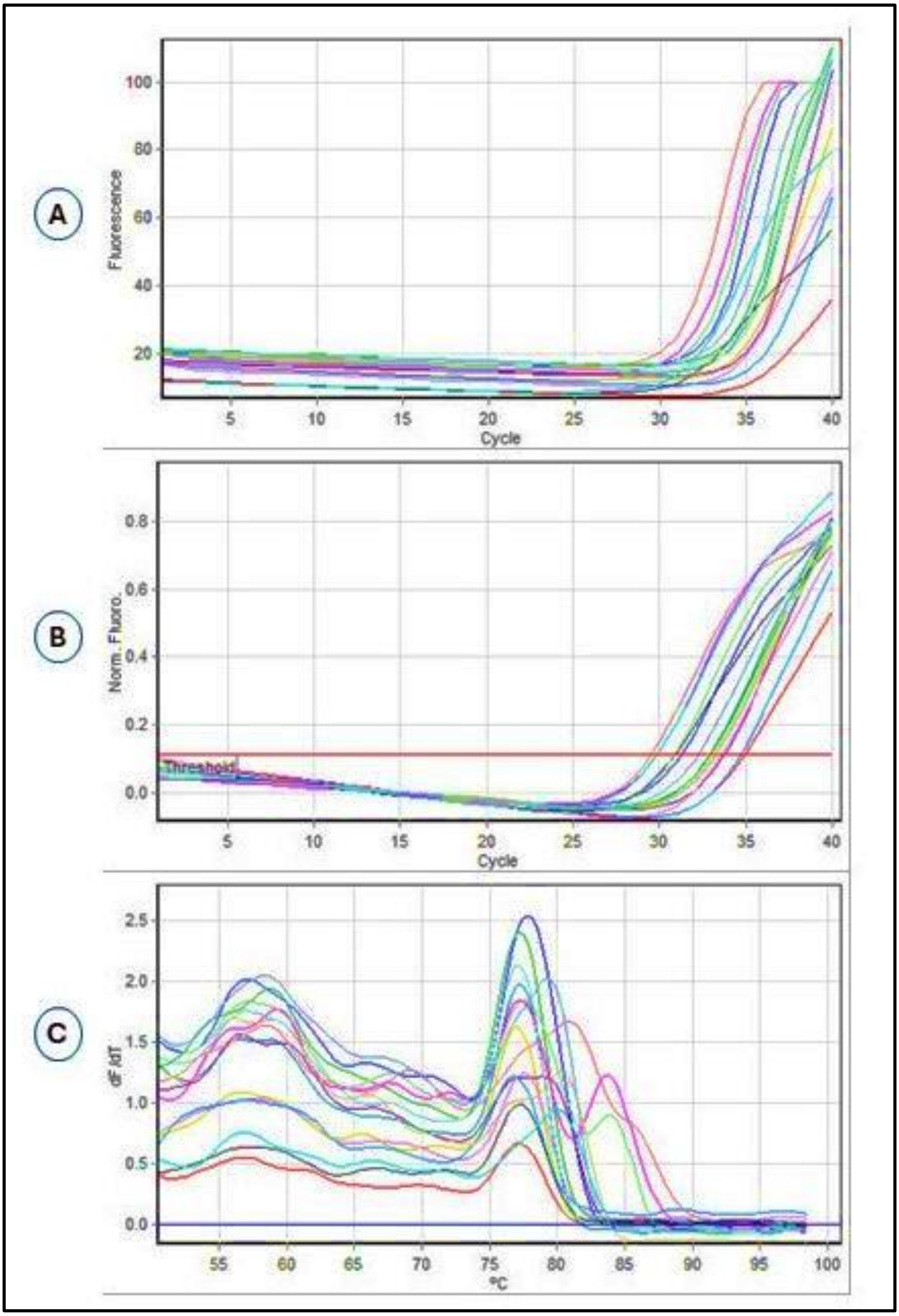

Figure S3: (A) The linear (upper), **(B)** log (lower) amplification curves representing the Ct values and **(C)** melting curves of PDK-2 gene in all experimental groups

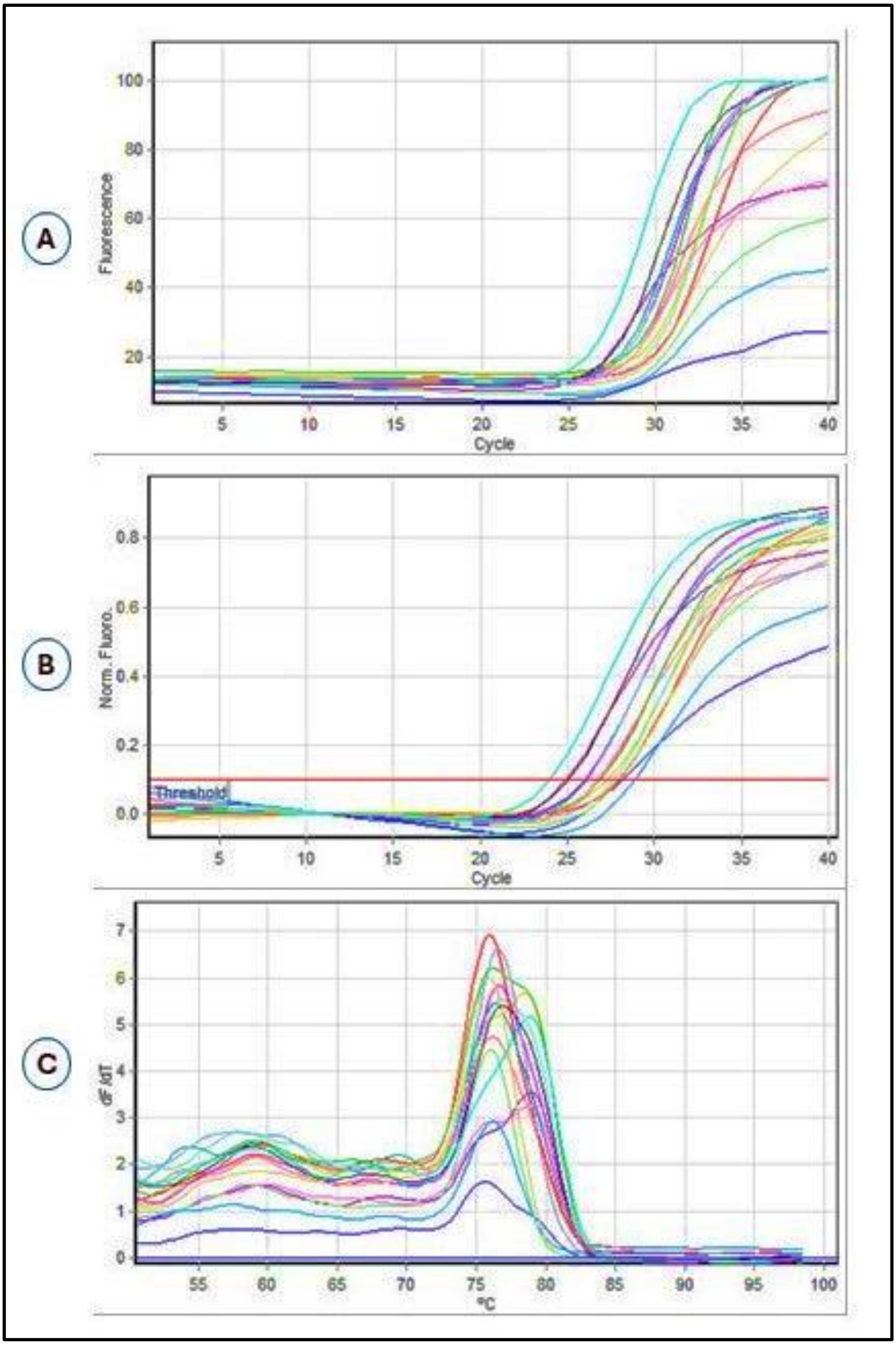

Figure S4: (A) The linear (upper), **(B)** log (lower) amplification curves representing the Ct values and **(C)** melting curves of PDK-3 gene in all experimental groups

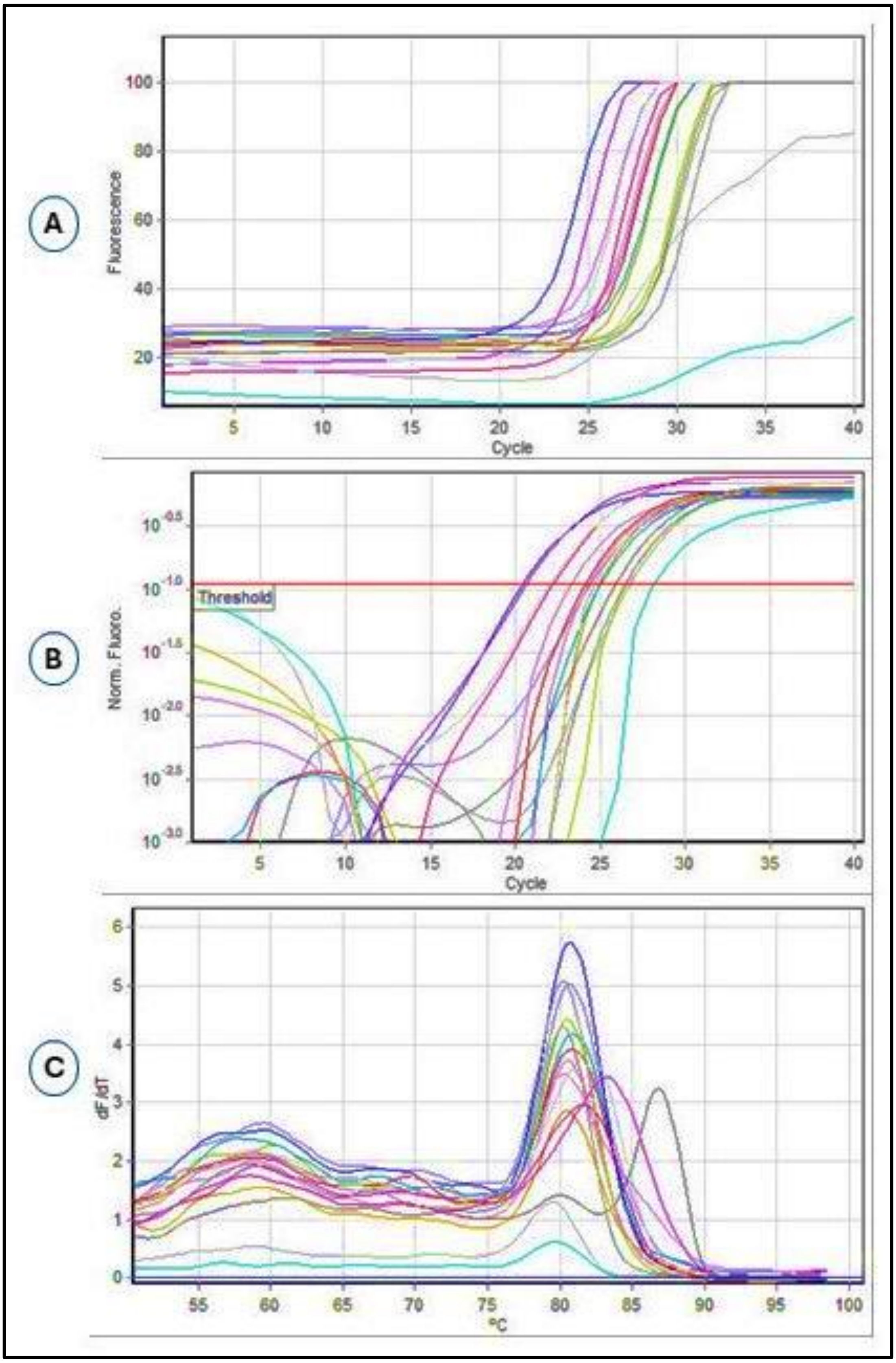

Figure S5: (A) The linear (upper), **(B)** log (lower) amplification curves representing the Ct values and **(C)** melting curves of PDK-4 gene in all experimental groups

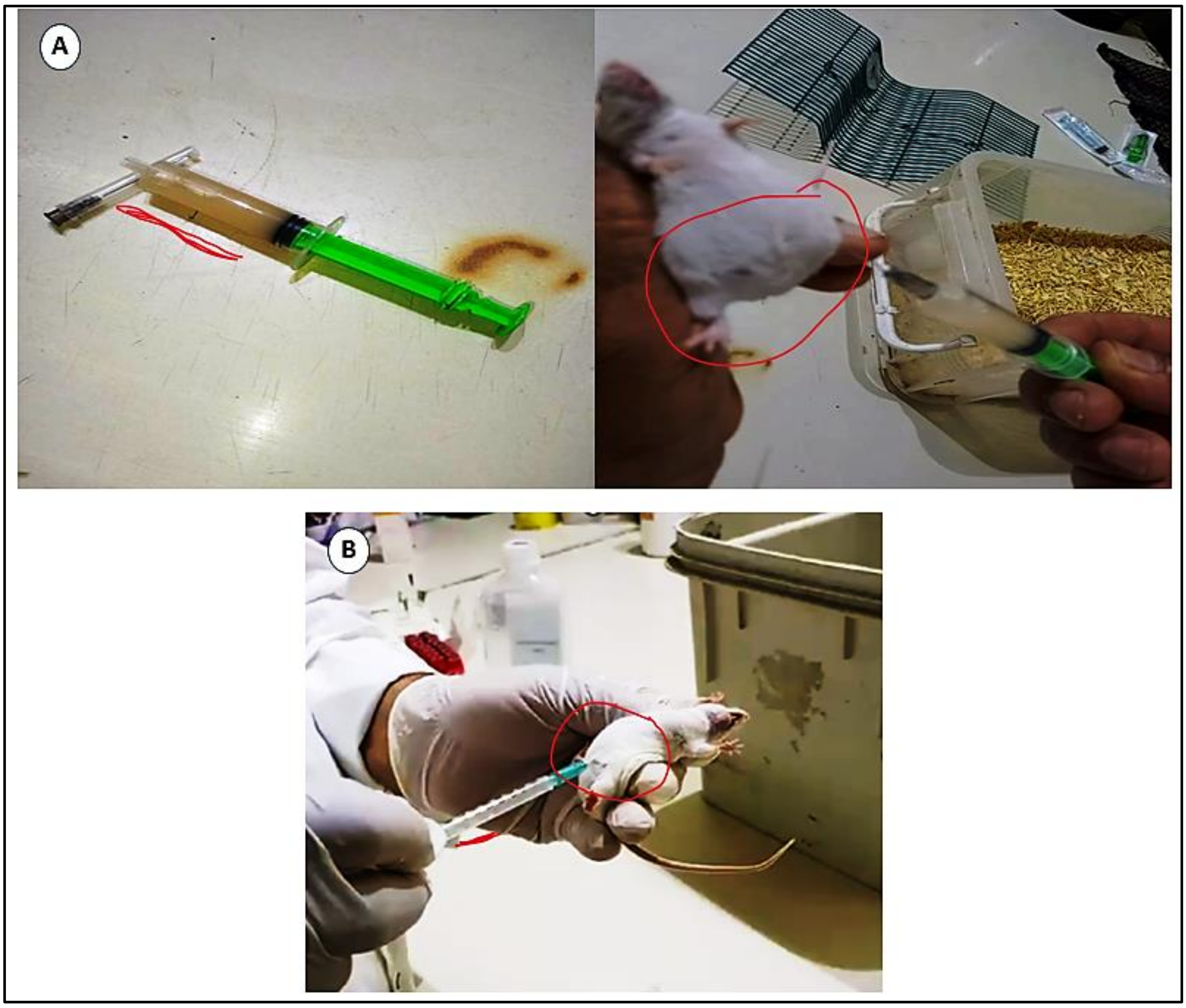

Figure S6: (A) Representative ascitic fluid collected from untreated EAC-bearing mice Gp1 with a picture of mice swelling during fluid withdraw from its abdominal cavity, **(B)** ascitic fluid collected from treated mice (EAC/Dox/DCA-PNPs) Gp10 showing a marked reduction in tumor burden and mice abdominal cavity.

Table S1: ADMET pharmacokinetics features.

Features	Name	DCA	Dox	PLGA	PVA
**Physicochemical properties**	**Smiles**	O=C(O)C(Cl)Cl	COc1cccc2c(O)c3c(O)c4c(c(O)c3c(O)c12)=C(O[C@H]1C[C@H](N)[C@H](O)[C@H](C)O1)C[C@](O)(C(=O)CO)C=4	O=C(O)CSc1ccccc1	CC=O
	**MW**	127.94	543.17	168.02	44.03
	**nHA**	2	12	2	1
	**nHD**	1	9	1	0
**Pharmacokinetics parameters**	**HIA**	0.505	0.751	0.011	0.006
	**PPB**	32.87%	91.29%	41.73%	31.3%
**Drug-likeness rules**	**Lipinski**	Accepted	Rejected	Accepted	Accepted
	**Pfizer**	Accepted	Accepted	Accepted	Accepted
	**GSK**	Accepted	Rejected	Accepted	Accepted
**Metabolism**	**CYP3A4-inh**	0.006	0.103	0.005	0.005
	**CYP3A4-sub**	0.056	0.128	0.157	0.227
**Toxicity prediction**	**CL**	6.035	9.566	7.429	7.937
	**T1/2**	0.895	0.847	0.864	0.757
	**hERG**	0.003	0.019	0.003	0.002
	**H-HT**	0.049	0.257	0.039	0.035
	**DILI**	0.029	0.964	0.016	0.026
	**Carcinogenicity**	0.029	0.776	0.011	0.012
**Stress response assays**	**SR-ARE**	0.585	0.813	0.114	0.384
	**SR-MMP**	0.018	0.959	0.017	0.745
	**SR-p53**	0.07	0.988	0.012	0.704

- Where, nHA, nHD: number of hydrogen bond acceptors and donors; HIA: human intestinal absorption; PPB: plasma protein binding; CYP: cytochrome P450 enzymes; CL: clearance; T1/2: half-life time; hERG: cardiac toxicity; H-HT,DILI: hepatotoxicity and drug-induced liver injury; SR: stress response; ARE: antioxidant response element; and MMP: mitochondrial membrane potential.

Table S2: Change in body weight of all groups.

Groups	Initial Body weight	Final body weight	B.W. %
**Ctrl**	21.5±0.93	24.975±0.66	0
**DCA Ctrl**	21.4 ±1.02	24.65±0.98	15.09
**DCA-PNPs Ctrl**	21.4± 1	24.39±0.83	13.89
**Dox Ctrl**	21.89 ±0.77	17.41±1.7***	20.45
**EAC**	22.9± 0.63	35.57±1.5****	55.21
**EAC/Dox**	22.9 ± 1.14	19.53±1.48^{++++}	14.58
**EAC/DCA**	22.5 ± 0.92	20.13±0.98^{++++}	10.5
**EAC/DCA-PNPs**	21.8 ± 0.7	17.62±0.77^{++++}	19.25
**EAC/Dox/DCA**	21.13 ± 0.84	16.515±0.54^{++++}	21.18
**EAC/Dox/DCA-PNPs**	20.16 ± 0.86	13.50±0.66^{++++}	33.05

Data are presented as mean ± SE n=4, (*p<0.0001) is deemed significant to Ctrl group and (+p<0.0001) denoting comparison to untreated EAC-bearing group.

Table S3: Change in ascitic volume and EAC cell count.

Groups	Ascitic volume (ml)	Viable cells count. (×10^6/ul)	Nonviable cells count. (×10^6/ul)	Total cells count. (×10^6/ul)
**EAC**	8.94±0.32	864±25.6	87.93±7.1	915.5±16.27
**EAC/Dox**	4.27±0.5+	366.25±17.4^	161.14±32.2'	459.5±12.08+
**EAC/DCA**	1.85±0.15+	19.73±0.76^	21.16±2.84'	40.89±2.08+
**EAC/DCA-PNPs**	0.94±0.02+	9.50±1.76^	6±1.29'	15.5±0.46+
**EAC/Dox/DCA**	1.082±0.09+	8.71±0.61^	8.75±1'	22.81±0.31+
**EAC/Dox/DCA-PNPs**	0.65±0.09+	2.06±0.49^	2.15±0.34'	4.21±0.13+

Data are presented as mean ± SE n=4, ^{+}p<0.0001 versus EAC bearing mice group and total cell number, ^p<0.0001 versus viable cells, and 'p<0.0001 versus non-viable cells.

Table S4: Effect of treatment on relative expression of cellular and mitochondrial PDK1-4 genes in all experimental groups.

Groups	Genes expression by fold change			
	PDK-1	PDK-2	PDK-3	PDK-4
	**Cellular Level**			
**Normal**	1 ± 0.1	1 ± 0.05	1 ± 0.36	1 ± 0.07
**EAC**<br>**% change**	5.8±0.4 [a]<br>480%	5.9±0.4 [a]<br>490%	5.5±0.9 [a]<br>450%	4.2±0.3 [a]<br>320%
**EAC/DOX**<br>**% change**	2.2±0.6 [b]<br>-62.06%	2.4±0.8 [b]<br>-59.32%	2.6±0.3 [b]<br>-52.72%	1.9±0.5 [b]<br>-54.76%
**EAC/DCA**<br>**% change**	2.1±0.2 [b]<br>-63.79%	2.1±0.3 [b]<br>-64.40%	2.5±0.1 [b]<br>-54.54%	1.8±0.1 [b]<br>-57.14%
**EAC/DCA-PNPs**<br>**% change**	1.9±1.4 [b]<br>-67.24%	2.1±0.5 [b]<br>-64.40%	2.4±0.2 [b]<br>-56.36%	1.6±0.5 [b]<br>-61.90%
**EAC/DCA/DOX**<br>**% change**	1.5±0.3 [b]<br>-74.13%	1.4±0.2 [b]<br>-76.27%	1.7±0.1 [b]<br>-69.09%	1.5±0.1 [b]<br>-64.28%
**EAC/DCA-NPs/DOX**<br>**% change**	1.3±0.5 [b]<br>-77.58%	.3±0.4 [b]<br>-77.96%	1.4±0.4 [b]<br>-74.54%	1.3±0.1 [b]<br>-69.04%
	**Mitochondrial level**			
**Normal**	1 ± 0.38	1 ± 0.52	1 ± 0.01	1 ± 0.03
**EAC**<br>**% change**	5.3±2.5[a]<br>430%	5.6±0.1 [a]<br>460%	5.7±0.4 [a]<br>470%	4.6±0.6 [a]<br>360%
**EAC/DOX**<br>**% change**	2.6±0.1[b]<br>-50.94%	3.5±0.1<br>-37.5%	3.8±0.1 [b]<br>-33.33%	2.6±0.1 [b]<br>-43.47%
**EAC/DCA**<br>**% change**	2.4±0.1 [b]<br>-54.71%	3.2±0.1<br>-42.85%	3.2±0.1 [b]<br>-43.85%	2.4±0.1 [b]<br>-47.82%
**EAC/DCA-PNPs**<br>**% change**	2.3±0.1 [b]<br>-56.60%	2.3±0.2<br>-58.92%	3.2±0.1 [b]<br>-43.85%	2.1±0.1 [b]<br>-54.34%
**EAC/DCA/DOX**<br>**% change**	1.8±0.1 [b]<br>-66.03%	1.8±0.5<br>-67.85%	2.1±0.1 [b]<br>-63.15%	1.7±0.1 [b]<br>-63.04%
**EAC/DCA-PNPs/DOX**<br>**% change**	1.3±0.1 [b]<br>-75.47%	1.3±0.1<br>-76.78%	1.3±0.1 [b]<br>-77.19%	1.4±0.2 [b]<br>-69.56%

- Data are presented as fold change ± SE n=4 . ([a]*$p<0.0001$) significance* value: versus normal control group, ([b]*$p<0.0001$) significance* value: versus EAC untreated group.

Table S5: Semi-quantitative histopathological grading of tumor cell infiltration in liver and kidney tissues.

Groups	Liver					Grade Mean ± SEM
	HPF 1	HPF 2	HPF 3	HPF 4	HPF 5	
**Percentage of tumor cells**						
**EAC-bearing mice**	3	3	2	2	3	**2.6±0.24**
**EAC/Dox**	3	2	1	3	1	**2±0.447**
**EAC/DCA**	2	2	3	2	1	**2±0.316**
**EAC/DCA-PNPs**	1	0	1	1	1	**0.8±0.2**[b]
**EAC/Dox/DCA**	1	0	1	1	1	**0.8±0.2**[b]
**EAC/Dox/DCA-PNPs**	0	1	0	0	1	**0.4±0.244**[b]
**Groups**	**Renal**					**Grade Mean ± SEM**
	**HPF 1**	**HPF 2**	**HPF 3**	**HPF 4**	**HPF 5**	
**Percentage of tumor cells**						
**EAC-bearing mice**	2	2	3	2	2	**2.6±0.244**
**EAC/Dox**	1	2	2	2	1	**1.6±0.244**[b]
**EAC/DCA**	1	2	1	2	1	**1.4±0.244**[b]
**EAC/DCA-PNPs**	1	0	1	1	1	**0.8±0.2**[b]
**EAC/Dox/DCA**	1	0	0	1	1	**0.6±0.245**[b]
**EAC/Dox/DCA-PNPs**	0	0	0	0	1	**0.2±0.2**[b]

Grading was performed based on the percentage of tumor cells observed in five random high-power fields (HPFs) per sample (n = 5 per group). Scores were assigned as follows: grade 0 = no lesion, grade 1 = mild lesion (<25% tissue involvement), grade 2 = moderate lesion (25–50% tissue involvement), and grade 3 = severe lesion (>50% tissue involvement). Mean ± SEM values were calculated for each group and used to generate the bar graphs shown in Figure 13 ([b]*p<0.0001) significance* value: versus EAC untreated group.
